# Association between changes in obesity- and lipid-related indices and the risk of cardiovascular disease: a prospective cohort study in Northwest China

**DOI:** 10.3389/fendo.2026.1975120

**Published:** 2026-10-09

**Authors:** Jinlong You, Wu Ding, Xue Wang, Jing Tang, Xiuqian Li, Rui Wang, Yuanyuan Li, Yana Bai, Minzhen Wang, Shan Zheng

**Affiliations:** Institute of Epidemiology and Statistics, School of Public Health, Lanzhou University, Lanzhou, China

**Keywords:** cardiovascular diseases, lipid metabolism, prospective cohort study, risk prediction, visceral adiposity

## Abstract

**Background:**

Cardiovascular disease (CVD) is a major global health burden. Traditional indicators inadequately capture CVD risk. This study evaluated the association between dynamic changes in novel obesity- and lipid-related indices and CVD incidence.

**Methods:**

A prospective cohort study was conducted with 19,315 CVD-free participants from the Jinchang Cohort in Northwest China. Changes in seven indices—WHtR, METS-VF, CVAI, BRI, TyG, TyG-WC, and TyG-BMI—from baseline to follow-up were classified via K-means clustering, and cumulative indices were calculated. Multivariable logistic and restricted cubic spline models assessed associations with incident CVD. Predictive performance was evaluated using AUC, NRI, and IDI.

**Results:**

Over a median follow-up of 66 months, 3,200 (16.57%) participants developed CVD. The highest change classes of all indices showed significantly increased CVD risk, with the strongest associations observed for TyG-BMI (OR: 3.988, 95% CI: 3.419–4.650), TyG-WC (OR: 3.797, 95% CI: 3.215–4.484), and CVAI (OR: 3.695, 95% CI: 3.085–4.426). A significant dose-response relationship was observed for cumulative indices. Changes in CVAI (AUC: 0.642) and cumulative METS-VF (AUC: 0.659) demonstrated the highest predictive ability. Adding these indices to a model of traditional risk factors significantly improved the AUC, NRI, and IDI for CVD prediction (all p < 0.001). Subgroup analyses were consistent.

**Conclusions:**

Longitudinal increases in obesity- and lipid-related indices may provide modest incremental value for CVD risk stratification when added to traditional risk factors, but their discriminative ability is moderate and further validation is required before clinical application.

## Introduction

Cardiovascular disease (CVD) is highly prevalent worldwide and is one of the leading causes of death globally, accounting for 35% of all deaths (1). The number of people with CVD increased from 271 million in 1990 to 523 million in 2019, and the number of deaths steadily increased by 53.7% during this period (2–4). At the same time, CVD is a major cause of premature deaths and rising health care costs (3, 5). At least 75% of the world’s CVD deaths occur in low- and middle-income countries, and China is one of the countries with the highest number of deaths due to CVD (6). According to the World Health Organization (WHO), CVD also accounts for the largest proportion of all deaths in China (7, 8). Uncontrolled hypertension is estimated to cause 750,000 CVD deaths annually in China, making it the most important cause of CVD deaths in the country (7, 9). In China, CVD is estimated to cost more than 40 billion dollars per year, about 4% of annual GDP, creating a serious economic burden (10). Therefore, CVD has undoubtedly become one of the major public health problems worldwide, and it is extremely important to assess the risk of CVD in the general population in order to provide effective prevention strategies.

It is well known that obesity has long been established as a traditional risk factor for CVD and has been recognized as a reliable predictor of ischemic heart disease, stroke incidence and premature mortality (11). A study from the China Health and Retirement Longitudinal Study (CHARLS) showed that the prevalence of obesity-associated hypertension among Chinese people aged 45 years and older was 22.7% (12). Previous studies have found a strong association between the development of CVD and insulin resistance (IR) (13, 14). Increased fat deposition triggers IR in all adipose and non-adipose tissues via endoplasmic reticulum stress, and the pancreas produces more insulin in order to compensate for the IR, which promotes fat synthesis and storage, creating a nasty cycle (15, 16). Two types of common obesity, central obesity and general obesity, are often assessed by waist circumference (WC) and body mass index (BMI), respectively (17). An epidemiologic survey from China showed that the prevalence of overweight or obesity among Chinese adults (≥18 years old) was 50.7% according to the BMI standard (18). However, a growing body of research suggests that BMI neither distinguishes between lean body mass and total body fat mass nor captures the distribution of body fat (19, 20). In addition, studies have found inconsistent associations between BMI and stroke (21–23), suggesting that BMI may not accurately assess CVD risk. Conversely, adipose tissue distribution, but not overall obesity, is an important determinant of CVD risk (24).

Radiologic imaging techniques can accurately assess abdominal obesity, but high costs and radiation hazards limit their use in large-scale epidemiologic investigations (25). Therefore, there is an urgent need to establish indicators that are closely related to abdominal obesity and are simple and effective. WC is a widely used indicator of central obesity, but it is deficient in distinguishing subcutaneous adipose tissue from visceral adipose tissue (26). Recently, several novel composite indicators were found to be independently associated with the incidence of CVD, including waist-to-height ratio (WHtR), metabolic score for visceral fat (METS-VF), Chinese visceral adiposity index (CVAI), and body roundness index (BRI) (17, 27, 28). In addition, the triglyceride-glucose (TyG) index has high sensitivity and specificity in the assessment of individual IR for the prediction of CVD (29, 30). Two previous studies from the National Health and Nutrition Examination Survey (NHANES) and the Kailuan cohort used the TyG index at baseline in combination with different indicators of obesity to form a new composite index, which has been shown to be strongly associated with CVD risk (31, 32). The above indicators take into account a variety of essential indicators of the human body, including WC, BMI, blood lipids, fasting plasma glucose, and other indicators, which may provide a more objective evaluation of the degree of obesity in the human body. However, previous studies have assessed the relationship with CVD risk mainly based on indicators measured or calculated at a single point in time, but these indicators are usually in a dynamic state of change over time. Because limited attention has been paid to the association between changes in obesity- and lipid-related indices and CVD, we explored the impact of changes in related indices on the risk of CVD based on a large prospective cohort study in Northwest China, including WHtR, METS-VF, CVAI, BRI,TyG, TyG-WC, and TyG-BMI. The main contributions of this study are summarized in **Box 1**.

box 1 **Contributions to the literature.**

This study moves beyond single-point measurements by demonstrating that longitudinal increases in a panel of obesity-lipid indices are associated with CVD risk, highlighting the importance of monitoring dynamic change.

It provides robust evidence from a large Northwest Chinese cohort on the superior performance of culturally-specific and composite indices like CVAI and TyG-BMI over traditional metrics, offering potentially useful markers for non-invasive risk assessment.

The findings underscore the incremental predictive value these indices add to conventional risk models, suggesting their integration could enhance early identification of high-risk individuals in public health and clinical practice, particularly in resource-limited settings.

## Methods

### Study subjects and design

This study is based on the Jinchang Cohort in Northwest China. The basic information of the Jinchang cohort has been described in detail in previous research (33, 34). 30,291 participants were ascertained with a 100% matching rate after pairing employee IDs and health insurance IDs between baseline and follow-ups. 8,248 patients with hypertension, 592 patients with coronary artery disease (CHD), 205 patients with stroke, 62 patients with malignant tumors at baseline, and 1169 participants lacking essential data on height, weight, blood lipids, blood pressure, fasting plasma glucose, and serum creatinine were excluded. Then, 700 participants who were considered to have anomalous metrics were excluded using the 3σ rule. 19,315 participants were ultimately included in this study (**Figure 1**). Prior to enrolling in the study, all participants signed a written informed consent form.

**Figure 1:**
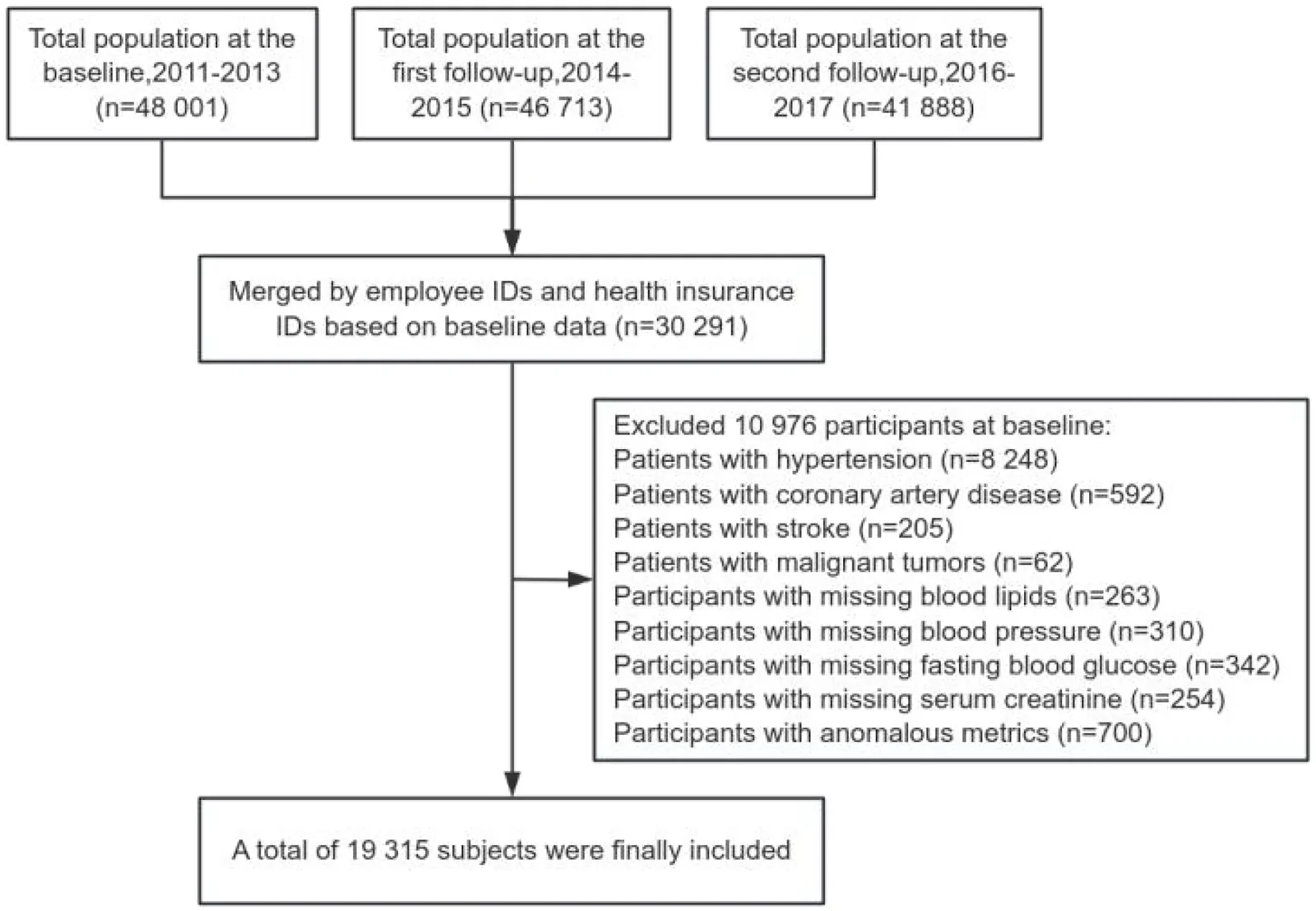
Flowchart of the inclusion and exclusion of participants.

All participants completed an epidemiological survey, physical examination, and biochemical tests. Epidemiological surveys were conducted by trained and assessed investigators using standardized and structured questionnaires to conduct face-to-face interviews focusing on general demographics (age, gender, education, occupation, and income), behavioral habits (smoking, alcohol consumption, exercise, and tea consumption), dietary habits (high salt, high fat, and high sugar diet), and personal history of diseases (diabetes, pre-diabetes, hyperuricemia, and other chronic diseases). Physical and biochemical examinations are performed by duly qualified medical professionals at tertiary hospitals affiliated with the enterprise. Blood and urine samples were collected after an overnight fast. Total cholesterol (TC), triglycerides (TG), high-density lipoprotein cholesterol (HDL-C), low-density lipoprotein cholesterol (LDL-C), uric acid (UA), fasting plasma glucose (FPG), serum creatinine (Scr) and other common clinical indicators in blood samples were measured with an automated measuring machine (Hitachi 7600-020, Kyoto, Japan).

### Assessment of exposures

The formulas of obesity- and lipid-related indices are shown in the **Supplementary Material** (35–40) (**Supplementary Table S1**). Height and weight were measured using an automated measuring machine (SK-X80/TCS-160D-W/H, Sonka, China) without shoes. WC was measured on the midaxillary line between the lowest border of the rib cage and the top of the iliac crest. We calculated the cumulative values of each indicator with reference to the accumulated TyG formula (41). The cumulative value of each indicator is defined as the average of the baseline value and the second follow-up value of that indicator, multiplied by the time interval (in years) between the two measurements. The changes in obesity- and lipid-related indices were classified using the K-means clustering method. K-means clustering is a centroid-based clustering method, which can minimize the sum of squared distances within each cluster (42). For each index, K-means clustering was performed separately using each participant’s baseline and follow-up values as a two-dimensional feature vector (baseline value, follow-up value), rather than the absolute change alone. This approach allowed the clustering to capture both the baseline level and the longitudinal change pattern of each index. Because the two features for a given index were measured on the same scale, no additional standardization was applied. Euclidean distance was used as the distance metric, and the algorithm was run with 50 random initializations and a maximum of 100 iterations to ensure convergence. The optimal number of clusters was determined using the elbow method and Gap statistics by evaluating k values from 1 to 10 and selecting the solution that balanced cluster compactness and interpretability. Accordingly, WHtR, METS-VF, BRI, and TyG were classified into three classes, while CVAI, TyG-WC, and TyG-BMI were classified into four classes (**Figure 2**; **Supplementary Figures S1**-**S6**).

**Figure 2:**
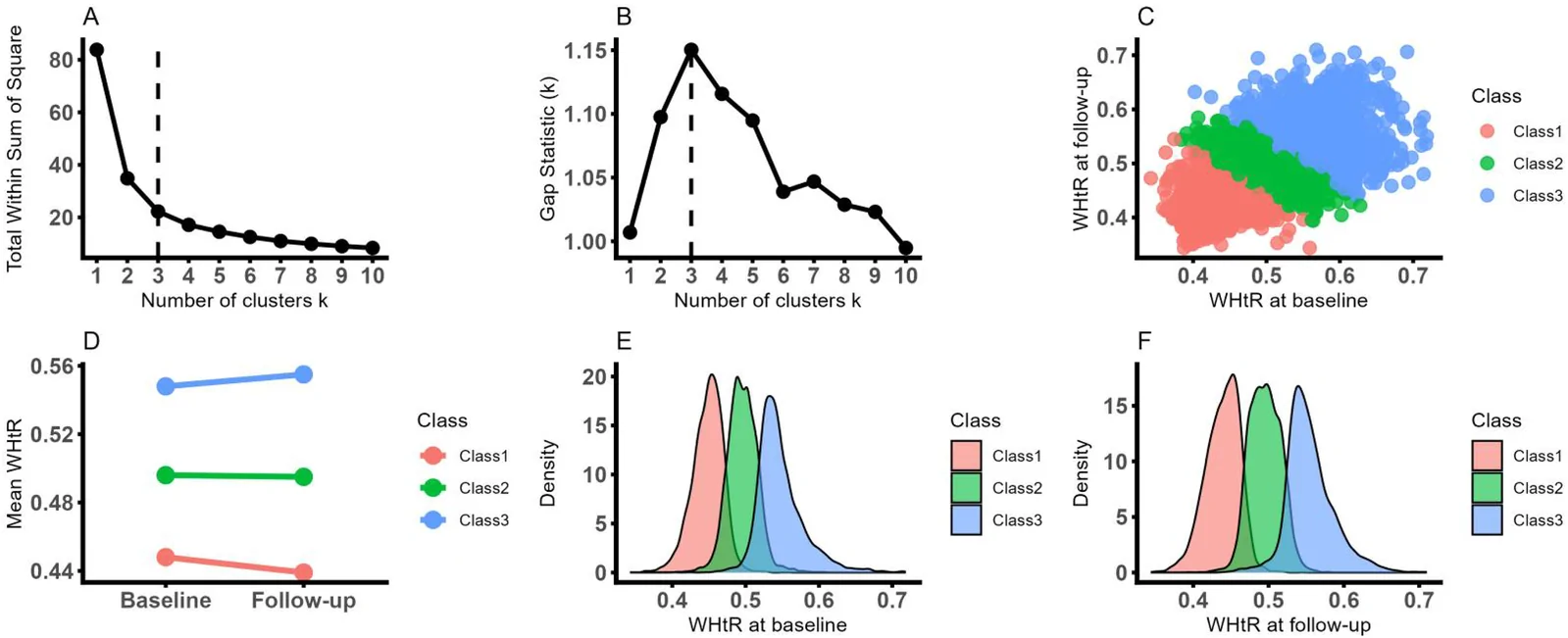
Clustering of the changes in WHtR from baseline to follow-up. **(A, B)** Determine the optimal number of clusters for the changes in WHtR using the Within-Cluster Sum of Squares (WSS) and Gap Statistics methods; **(C)** Changes in WHtR classified into three classes using the K-means algorithm; **(D)** Data visualization of mean WHtR across different classes; **(E, F)** Distribution of WHtR at baseline or follow-up.

### Outcomes of interest

The emergence of the study’s findings was defined as the first event of nonfatal CVD and was coded according to the ICD-10, including hypertension (ICD-10: I10-I15), CHD (ICD-10: I20-I25), and stroke (ICD-10: I60-I65). The inclusion of new-onset hypertension in the composite CVD outcome was based on the ICD-10 classification, in which essential hypertension (I10-I15) is categorized under Chapter IX, ‘Diseases of the circulatory system’, together with CHD and stroke. To evaluate whether this broad definition influenced our findings, we performed a sensitivity analysis restricting the outcome to hard cardiovascular events, defined exclusively as incident nonfatal CHD and stroke, excluding isolated hypertension. The results showed that the positive associations between the dynamic changes in all seven obesity- and lipid-related indices and CVD risk remained statistically significant after full adjustment (**Supplementary Table S6**). Hypertension was defined as systolic blood pressure ≥ 140 mmHg (1 mmHg = 0.133 kPa) and/or diastolic blood pressure ≥ 90 mmHg or a self-reported diagnosis of hypertension accompanied by a certificate of diagnosis in hospitals of the second level or above (43). CHD was defined as a self-reported diagnosis of CHD accompanied by a certificate of diagnosis in hospitals of the second level or above, and/or patients diagnosed with CHD in previous hospitalized medical records. Stroke was defined in the same way as CHD.

### Assessment of covariates

High salt, high fat, and high sugar diets were defined as > 6 g/d of salt, > 30 g/d of cooking oil, > 50 g/d of sugar, respectively. Participants who had smoked at least one cigarette per day for more than 6 months or those who had quit smoking for less than 6 months were defined as smoking, and those who had quit smoking for more than 6 months were defined as smoking cessation. Participants who drank spirits, beer, or wine at least one time per week for more than 6 months or those who had quit drinking for less than 6 months were defined as drinking, and those who had quit drinking for more than 6 months were defined as drinking cessation. Physical exercise included never, occasional, and regular. Participants who exercised one to three times per week for more than 30 minutes per session were defined as occasional exercise, and those who exercised at least three times per week for more than 30 minutes per session were defined as regular exercise. Tea consumption included never, occasional, and regular. Participants who drank tea one to three times per week for more than 6 months were defined as occasional tea consumption, and those who drank tea at least three times per week for more than 6 months were defined as regular tea consumption. Family history of CVD: at least one of the first-degree relatives of the study participants had a cardiovascular type (hypertension, stroke, or CHD) of disease. Diabetes was defined as FPG ≥ 7.0 mmol/L or a self-reported diagnosis of diabetes accompanied by a certificate of diagnosis from a hospital of the second level or higher, and participants with FPG between 5.6 and 6.9 mmol/L were determined to be pre-diabetic (44). Hyperuricemia was defined as a fasting serum UA level of more than 420μmol/L after 2 consecutive days of a normal diet (45). Diagnostic findings of fatty liver disease based on abdominal ultrasound, and other chronic diseases were identified from epidemiological surveys and/or previous hospitalisation medical records.

### Statistical analysis

Missing data and outlier handling: Participants with missing essential data on height, weight, blood lipids, blood pressure, fasting plasma glucose, or serum creatinine were excluded during cohort construction (n = 1,169), as described in the Study subjects and design section. After these exclusions, all variables included in the final multivariable models were complete, and no additional missing data were present in the 19,315 participants who constituted the analytical cohort. Therefore, complete-case analysis was used, and no imputation was performed. For continuous variables, the 3σ rule was applied to identify anomalous metrics, and participants with values beyond mean ± 3 standard deviations for any key anthropometric, biochemical, or blood pressure variable were excluded (n = 700), as also described in the Study subjects and design section. A sensitivity analysis excluding these participants yielded results consistent with the main findings. Categorical variables were summarized using frequencies and proportions, while continuous variables were expressed as mean ± standard deviation. Comparisons between groups for continuous and categorical variables were conducted using Student’s t-test and Pearson’s chi-square test, respectively. The cumulative incidence of CVD across quartile groups was compared using Kaplan-Meier curves with log-rank tests.

In this study, the temporal information from the two follow-up waves was incorporated into the exposure definition in two ways. First, K-means clustering was performed on the two-dimensional feature vector consisting of each participant’s baseline and follow-up values, so that the resulting change classes jointly reflected the starting level and the magnitude and direction of change over time. Second, the cumulative value of each index was calculated as the average of the baseline and follow-up values multiplied by the time interval between the two measurements, thereby explicitly incorporating the duration of exposure. Because the exposure contrast was defined at the level of change classes and cumulative exposure rather than at a single time point, logistic regression was used to estimate the association between these structured exposure categories and the binary outcome of incident CVD ascertained at the second follow-up. The results were shown as odds ratios (ORs) and 95% confidence intervals (CIs). Three models were constructed: Model 1 adjusted for gender and age; Model 2 additionally adjusted for nationality, occupation, education, marital status, per capita monthly income, smoking status, alcohol consumption, physical exercise, tea consumption, high salt diet, high fat diet, and high sugar diet. Model 3 additionally adjusted for diabetes or pre-diabetes, hyperuricemia, family history of CVD, Scr, and AST/ALT. P values for trend were calculated using the median value in each quartile. The OR of CVD incidence per 1 SD increase in the cumulative value for each indicator was also estimated. Restricted cubic spline (RCS) regression model was employed to analyze the dose-response relationship between cumulative value for each indicator and CVD. Nodes were established at the percentile of the independent variables (5%, 35%, 65%, and 95%). To evaluate the predictive performance of obesity- and lipid-related indices for incident CVD, a receiver operating characteristic (ROC) curve analysis was conducted. Pairwise comparisons using Delong’s test were used to identify indices with superior predictive performance. The area under the curve (AUC), net reclassification improvement (NRI), and integrated discrimination improvement (IDI) were conducted to investigate the additional contribution of each indicator to new-onset CVD in the context of traditional cardiovascular risk factors. Furthermore, we conducted subgroup analyses to explore the potential relationship between obesity- and lipid-related indices and CVD under different stratifications such as by gender, age, nationality, occupation, education, marital status, per capita monthly income, smoking status, alcohol consumption, physical exercise, tea consumption, high salt diet, high fat diet, high sugar diet, diabetes or pre-diabetes, hyperuricemia, and family history of CVD.

All statistical tests were two-sided and p < 0.05 was considered statistically significant. R software version 4.3.2 (R foundation, Vienna, Austria) and SPSS software version 29.0 (IBM SPSS Inc., Chicago, IL, USA) were used for all statistical analysis.

## Results

### Baseline characteristics of the participants

The baseline characteristics of 19,315 participants are summarized in **Table 1**. Compared to non-CVD individuals, the CVD group exhibited significantly older age (47.50 vs. 41.20 years), higher male predominance (63.34% vs. 57.49%), and lower educational attainment (41.19% vs. 26.99% with middle school or below). Lifestyle risk factors, including smoking, alcohol use, and high-salt/fat/sugar diets, were more prevalent in CVD participants (all p < 0.001). Comorbidities such as diabetes/pre-diabetes (25.53% vs. 14.20%) and fatty liver disease (26.03% vs. 17.09%) were markedly elevated in the CVD group. Adverse metabolic profiles were observed, including higher lipid levels (TG: 2.08 vs. 1.75 mmol/L), elevated blood pressure (SBP: 121.20 vs. 113.21 mmHg), and increased adiposity indices (BMI: 23.85 vs. 22.55 kg/m²). No significant differences were noted in nationality or family CVD history (p > 0.05). These findings underscore distinct demographic, behavioral, and metabolic disparities in CVD development.

**Table 1:** Baseline characteristics of new-onset CVD and non-CVD participants.

Characteristic	Total (n=19315)	Non-CVD (n=16115)	CVD (n=3200)	*p*
Gender, n(%)				<0.001
Male	11292 (58.46)	9265 (57.49)	2027 (63.34)	
Female	8023 (41.54)	6850 (42.51)	1173 (36.66)	
Age, years	42.24 (10.77)	41.20 (10.17)	47.50 (12.08)	<0.001
Nationality, n(%)				0.329
Han nationality	18716 (96.90)	15606 (96.84)	3110 (97.19)	
Others	599 (3.10)	509 (3.16)	90 (2.81)	
Occupation, n(%)				<0.001
Leader	2061 (10.67)	1703 (10.57)	358 (11.19)	
Technicist	996 (5.16)	867 (5.38)	129 (4.03)	
Worker	15825 (81.93)	13238 (82.15)	2587 (80.84)	
Logistics staff	433 (2.24)	307 (1.91)	126 (3.94)	
Education, n(%)				<0.001
Middle school or below	5667 (29.34)	4349 (26.99)	1318 (41.19)	
High school or equivalent	5909 (30.59)	4982 (30.92)	927 (28.97)	
College or above	7739 (40.07)	6784 (42.10)	955 (29.84)	
Marital status, n(%)				<0.001
Single	2208 (11.43)	1950 (12.10)	258 (8.06)	
Married	17107 (88.57)	14165 (87.90)	2942 (91.94)	
Per capita monthly income (¥), n(%)				0.091
< ¥2000	9946 (51.49)	8254 (51.22)	1692 (52.88)	
≥ ¥2000	9369 (48.51)	7861 (48.78)	1508 (47.12)	
Smoking status, n(%)				<0.001
No	10983 (56.86)	9292 (57.66)	1691 (52.84)	
Yes	7247 (37.52)	6011 (37.30)	1236 (38.62)	
Smoking cessation	1085 (5.62)	812 (5.04)	273 (8.53)	
Alcohol consumption, n(%)				<0.001
No	14833 (76.80)	12527 (77.74)	2306 (72.06)	
Yes	3879 (20.08)	3140 (19.48)	739 (23.09)	
Drinking cessation	603 (3.12)	448 (2.78)	155 (4.84)	
Physical exercise, n(%)				<0.001
Never	2896 (14.99)	2479 (15.38)	417 (13.03)	
Occasionally	8540 (44.21)	7237 (44.91)	1303 (40.72)	
Regularly	7879 (40.79)	6399 (39.71)	1480 (46.25)	
Tea consumption, n(%)				<0.001
Never	9361 (48.47)	7902 (49.04)	1459 (45.59)	
Occasionally	9796 (50.72)	8091 (50.21)	1705 (53.28)	
Regularly	157 (0.81)	121 (0.75)	36 (1.12)	
High salt diet, n(%)	4149 (21.48)	3343 (20.74)	806 (25.19)	<0.001
High fat diet, n(%)	3565 (18.46)	2878 (17.86)	687 (21.47)	<0.001
High sugar diet, n(%)	3835 (19.86)	3130 (19.42)	705 (22.03)	0.001
Diabetes or pre-diabetes, n(%)	3106 (16.08)	2289 (14.20)	817 (25.53)	<0.001
Hyperuricemia, n(%)	1864 (9.65)	1441 (8.94)	423 (13.22)	<0.001
Family history of CVD, n(%)	7185 (37.20)	5990 (37.17)	1195 (37.34)	0.869
Fatty liver diseases, n(%)	3587 (18.57)	2754 (17.09)	833 (26.03)	<0.001
Chronic respiratory diseases, n(%)	4111 (21.28)	3423 (21.24)	688 (21.50)	0.762
Chronic digestive diseases, n(%)	6192 (32.06)	4967 (30.82)	1225 (38.28)	<0.001
Genitourinary Diseases, n(%)	3131 (16.21)	2578 (16.00)	553 (17.28)	0.076
TC, mmol/L	4.60 ± 0.85	4.57 ± 0.83	4.76 ± 0.90	<0.001
TG, mmol/L	1.80 ± 1.43	1.75 ± 1.36	2.08 ± 1.72	<0.001
HDL-C, mmol/L	1.39 ± 0.35	1.40 ± 0.35	1.36 ± 0.35	<0.001
LDL-C, mmol/L	3.00 ± 0.71	2.97 ± 0.70	3.12 ± 0.74	<0.001
FPG, mmol/L	5.13 ± 1.13	5.07 ± 1.05	5.42 ± 1.44	<0.001
Scr, μmol/L	68.67 ± 13.66	68.70 ± 13.67	68.53 ± 13.61	0.532
AST/ALT	1.19 ± 0.44	1.19 ± 0.44	1.17 ± 0.43	0.009
SBP, mmHg	114.53 ± 12.93	113.21 ± 12.70	121.20 ± 11.99	<0.001
DBP, mmHg	73.48 ± 8.00	72.73 ± 7.79	77.27 ± 7.93	<0.001
WC, cm	82.50 ± 8.50	82.04 ± 8.42	84.84 ± 8.51	<0.001
BMI, kg/m^2^	22.76 ± 2.98	22.55 ± 2.93	23.85 ± 3.01	<0.001
WHtR	0.49 ± 0.04	0.49 ± 0.04	0.50 ± 0.04	<0.001
METS-VF	5.64 ± 0.47	5.60 ± 0.46	5.85 ± 0.43	<0.001
CVAI	75.04 ± 39.64	71.77 ± 39.30	91.51 ± 37.22	<0.001
BRI	3.22 ± 0.82	3.16 ± 0.81	3.48 ± 0.86	<0.001
TyG	8.71 ± 0.62	8.67 ± 0.61	8.89 ± 0.64	<0.001
TyG-WC	720.27 ± 104.73	713.29 ± 103.27	755.39 ± 104.94	<0.001
TyG-BMI	198.88 ± 33.94	196.17 ± 33.27	212.52 ± 33.97	<0.001

TC, total cholesterol; TG, triglyceride; HDL-C, high-density lipoprotein cholesterol; LDL-C, low-density lipoprotein cholesterol; FPG, fasting plasma glucose; Scr, serum creatinine; AST/ALT, aspartate aminotransferase/alanine aminotransferase; SBP, systolic blood pressure; DBP, diastolic blood pressure; WC, waist circumference; BMI, body mass index; WHtR, waist-to-height ratio; METS-VF, metabolic score for visceral fat; CVAI, Chinese visceral adiposity index; BRI, body roundness index; TyG, triglyceride-glucose; TyG-WC, TyG-waist circumference; TyG-BMI, TyG-body mass index.

### Cumulative incidence of CVD

During a median follow-up period of 66 months, 3200 (16.57%) participants experienced incident CVD. Kaplan–Meier curves for incident CVD by quartiles of obesity- and lipid-related indices are presented in **Figure 3**. Higher baseline WHtR, METS-VF, CVAI, BRI, TyG, TyG-WC, and TyG-BMI were correlated with an elevated risk of CVD (all log-rank tests p < 0.001).

**Figure 3:**
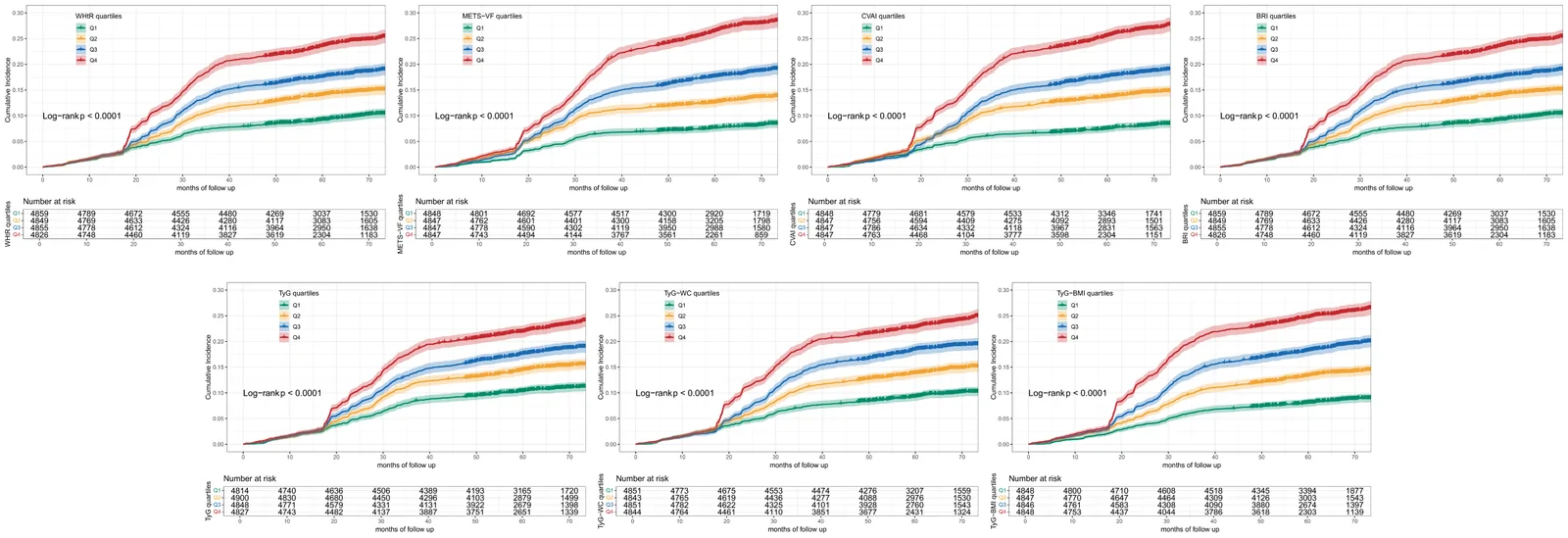
Kaplan-Meier curves for the cumulative incidence of CVD. WHtR, waist-to-height ratio; METS-VF, metabolic score for visceral fat; CVAI, Chinese visceral adiposity index; BRI, body roundness index; TyG, triglyceride-glucose; TyG-WC, TyG-waist circumference; TyG-BMI, TyG-body mass index.

### CVD risk analysis

As shown in **Table 2**, participants were grouped based on changes in obesity- and lipid-related indices using K-means clustering. In the fully adjusted model (Model 3), those in the highest change class (Class 3 or Class 4) of each indicator had significantly higher CVD risk compared to those in the lowest class (Class 1, reference). Positive associations were observed for changes in WHtR (Class 3 OR: 2.110, 95% CI: 1.867–2.385), METS-VF (Class 3 OR: 2.332, 95% CI: 2.004–2.713), CVAI (Class 4 OR: 3.695, 95% CI: 3.085–4.426), BRI (Class 3 OR: 2.127, 95% CI: 1.883–2.403), TyG (Class 3 OR: 1.897, 95% CI: 1.677–2.146), TyG-WC (Class 4 OR: 3.797, 95% CI: 3.215–4.484), and TyG-BMI (Class 4 OR: 3.988, 95% CI: 3.419–4.650). Notably, the ORs increased significantly with higher change classes for all indices (all p for trend < 0.05). As shown in **Supplementary Table S2**, similar results were also observed in the cumulative values of each indicator. Using the first quartile as reference, a stepwise increase of new-onset CVD was observed with the increased cumulative values of each indicator (all p for trend < 0.05). These results remained similar after adjusting for potential confounders.

**Table 2:** Associations of changes in obesity- and lipid-related indices with CVD incidence.

	Model 1^a^		Model 2^b^		Model 3^c^	
	OR (95% CI)	*p*	OR (95% CI)	*p*	OR (95% CI)	*p*
WHtR						
Class 1	Ref.		Ref.		Ref.	
Class 2	1.550 (1.398 - 1.719)	<0.001	1.534 (1.383 - 1.702)	<0.001	1.445 (1.300 ~ 1.605)	<0.001
Class 3	2.428 (2.157 - 2.732)	<0.001	2.381 (2.114 - 2.681)	<0.001	2.110 (1.867 ~ 2.385)	<0.001
*P* for trend	<0.001		<0.001		<0.001	
METS-VF						
Class 1	Ref.		Ref.		Ref.	
Class 2	1.664 (1.442 - 1.920)	<0.001	1.692 (1.464 - 1.955)	<0.001	1.583 (1.369 ~ 1.831)	<0.001
Class 3	2.657 (2.295 - 3.076)	<0.001	2.670 (2.302 - 3.096)	<0.001	2.332 (2.004 ~ 2.713)	<0.001
*P* for trend	<0.001		<0.001		<0.001	
CVAI						
Class 1	Ref.		Ref.		Ref.	
Class 2	1.771 (1.531 - 2.049)	<0.001	1.771 (1.529 - 2.050)	<0.001	1.702 (1.468 ~ 1.974)	<0.001
Class 3	2.893 (2.477 - 3.379)	<0.001	2.865 (2.450 - 3.349)	<0.001	2.600 (2.215 ~ 3.052)	<0.001
Class 4	4.409 (3.712 - 5.238)	<0.001	4.332 (3.643 - 5.152)	<0.001	3.695 (3.085 ~ 4.426)	<0.001
*P* for trend	<0.001		<0.001		<0.001	
BRI						
Class 1	Ref.		Ref.		Ref.	
Class 2	1.567 (1.418 - 1.732)	<0.001	1.548 (1.400 - 1.712)	<0.001	1.452 (1.311 ~ 1.608)	<0.001
Class 3	2.456 (2.184 - 2.762)	<0.001	2.406 (2.138 - 2.708)	<0.001	2.127 (1.883 ~ 2.403)	<0.001
*P* for trend	<0.001		<0.001		<0.001	
TyG						
Class 1	Ref.		Ref.		Ref.	
Class 2	1.626 (1.480 - 1.786)	<0.001	1.625 (1.479 - 1.786)	<0.001	1.506 (1.368 ~ 1.658)	<0.001
Class 3	2.287 (2.041 - 2.563)	<0.001	2.279 (2.031 - 2.558)	<0.001	1.897 (1.677 ~ 2.146)	<0.001
*P* for trend	<0.001		<0.001		<0.001	
TyG-WC						
Class 1	Ref.		Ref.		Ref.	
Class 2	1.754 (1.547 - 1.987)	<0.001	1.738 (1.533 - 1.971)	<0.001	1.674 (1.475 ~ 1.900)	<0.001
Class 3	2.728 (2.374 - 3.135)	<0.001	2.689 (2.338 - 3.091)	<0.001	2.426 (2.101 ~ 2.801)	<0.001
Class 4	4.589 (3.925 - 5.365)	<0.001	4.503 (3.848 - 5.271)	<0.001	3.797 (3.215 ~ 4.484)	<0.001
*P* for trend	<0.001		<0.001		<0.001	
TyG-BMI						
Class 1	Ref.		Ref.		Ref.	
Class 2	1.669 (1.471 - 1.895)	<0.001	1.671 (1.471 - 1.897)	<0.001	1.624 (1.428 ~ 1.846)	<0.001
Class 3	2.627 (2.314 - 2.983)	<0.001	2.617 (2.303 - 2.974)	<0.001	2.447 (2.144 ~ 2.793)	<0.001
Class 4	4.531 (3.925 - 5.230)	<0.001	4.507 (3.899 - 5.209)	<0.001	3.988 (3.419 ~ 4.650)	<0.001
*P* for trend	<0.001		<0.001		<0.001	

WHtR, waist-to-height ratio; METS-VF, metabolic score for visceral fat; CVAI, Chinese visceral adiposity index; BRI, body roundness index; TyG, triglyceride-glucose; TyG-WC, TyG-waist circumference; TyG-BMI, TyG-body mass index; OR, odds ratio; CI, confidence interval.

^a^ Adjusted for gender and age;.

^b^ Adjusted for gender, age, nationality, occupation, education, marital status, per capita monthly income, smoking status, alcohol consumption, physical exercise, tea consumption, high salt diet, high fat diet, and high sugar diet.

^c^ Adjusted for variables in Model 2 and diabetes or pre-diabetes, hyperuricemia, family history of CVD, Scr, and AST/ALT.

### Dose–response relationship analysis

As shown in **Figure 4**, the RCS models revealed significant nonlinear associations between cumulative CVAI, cumulative BRI, cumulative TyG, and cumulative TyG-WC with the risk of incident CVD in all three covariate-adjusted models (all p for nonlinear < 0.05).

**Figure 4:**
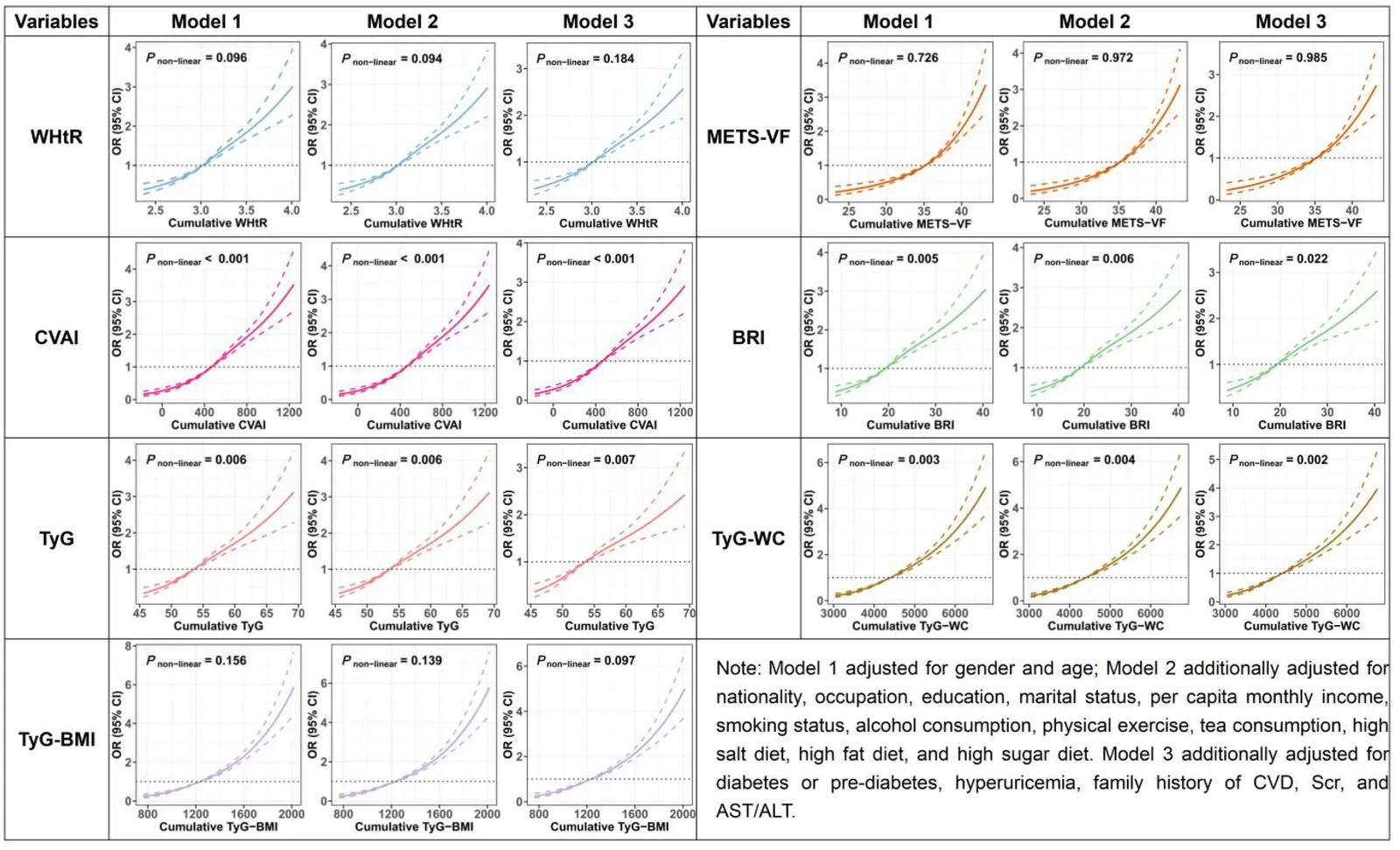
The dose-response relationship between the cumulative value of obesity- and lipid-related indices and CVD. WHtR, waist-to-height ratio; METS-VF, metabolic score for visceral fat; CVAI, Chinese visceral adiposity index; BRI, body roundness index; TyG, triglyceride-glucose; TyG-WC, TyG-waist circumference; TyG-BMI, TyG-body mass index.

### Prediction and reclassification

The predictive ability of obesity- and lipid-related indices for new-onset CVD was evaluated using ROC curve analysis. As shown in **Figure 5**, changes in CVAI (AUC: 0.642, 95% CI: 0.632-0.651) and cumulative METS-VF (AUC: 0.659, 95% CI: 0.649-0.670) demonstrated the highest predictive performance for new-onset CVD. Furthermore, **Table 3** shows that there were no statistically significant differences in AUC values between changes in CVAI and changes in TyG-BMI, or between cumulative METS-VF and cumulative CVAI, indicating that both had relatively better discriminatory ability among the evaluated indices, although overall performance remained moderate.

**Figure 5:**
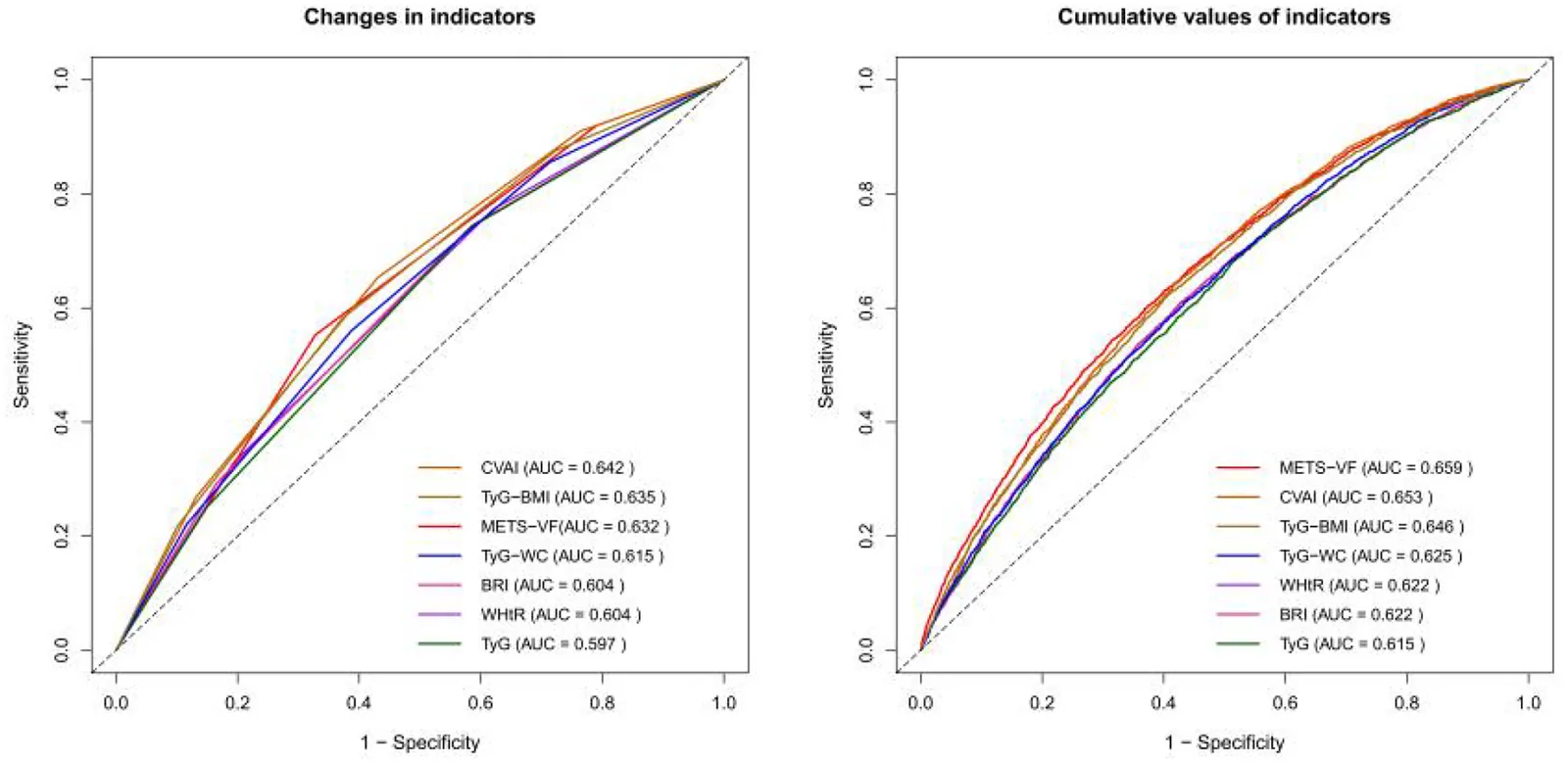
The ROC curves for obesity- and lipid-related indices predicting CVD. WHtR, waist-to-height ratio; METS-VF, metabolic score for visceral fat; CVAI, Chinese visceral adiposity index; BRI, body roundness index; TyG, triglyceride-glucose; TyG-WC, TyG-waist circumference; TyG-BMI, TyG-body mass index; AUC, area under the curve.

**Table 3:** The comparison of AUC among obesity- and lipid-related indices.

Changes in indicators	AUC (95% CI)	*p*	Cumulative values of indicators	AUC (95% CI)	*p*
CVAI	0.642 (0.632 - 0.651)	Ref.	METS-VF	0.659 (0.649 - 0.670)	Ref.
TyG-BMI	0.635 (0.625 - 0.645)	0.089	CVAI	0.653 (0.643 - 0.663)	0.085
METS-VF	0.632 (0.623 - 0.642)	0.022	TyG-BMI	0.646 (0.636 - 0.656)	0.005
TyG-WC	0.615 (0.605 - 0.625)	<0.001	TyG-WC	0.625 (0.614 - 0.635)	<0.001
BRI	0.604 (0.594 - 0.614)	<0.001	WHtR	0.622 (0.611 - 0.632)	<0.001
WHtR	0.604 (0.594 - 0.614)	<0.001	BRI	0.622 (0.611 - 0.632)	<0.001
TyG	0.597 (0.587 - 0.607)	<0.001	TyG	0.615 (0.604 - 0.625)	<0.001

WHtR, waist-to-height ratio; METS-VF, metabolic score for visceral fat; CVAI, Chinese visceral adiposity index; BRI, body roundness index; TyG, triglyceride-glucose; TyG-WC, TyG-waist circumference; TyG-BMI, TyG-body mass index; AUC, area under the curve; CI, confidence interval.

To assess the additional prognostic value of obesity- and lipid-related indices beyond traditional risk factors, we established a baseline model incorporating gender, age, nationality, occupation, education, marital status, per capita monthly income, smoking status, alcohol consumption, physical exercise, tea consumption, high salt diet, high fat diet, high sugar diet, diabetes or pre-diabetes, hyperuricemia, family history of CVD, TC, LDL-C, Scr, and AST/ALT. As shown in **Figure 6** and **Table 4**, the inclusion of obesity- and lipid-related indices significantly enhanced the predictive performance of the baseline model for new-onset CVD, as evidenced by statistically significant increases in AUC values (all p < 0.05 by Delong’s test). Moreover, adding obesity- and lipid-related indices significantly improved the NRI and IDI for new-onset CVD.

**Figure 6:**
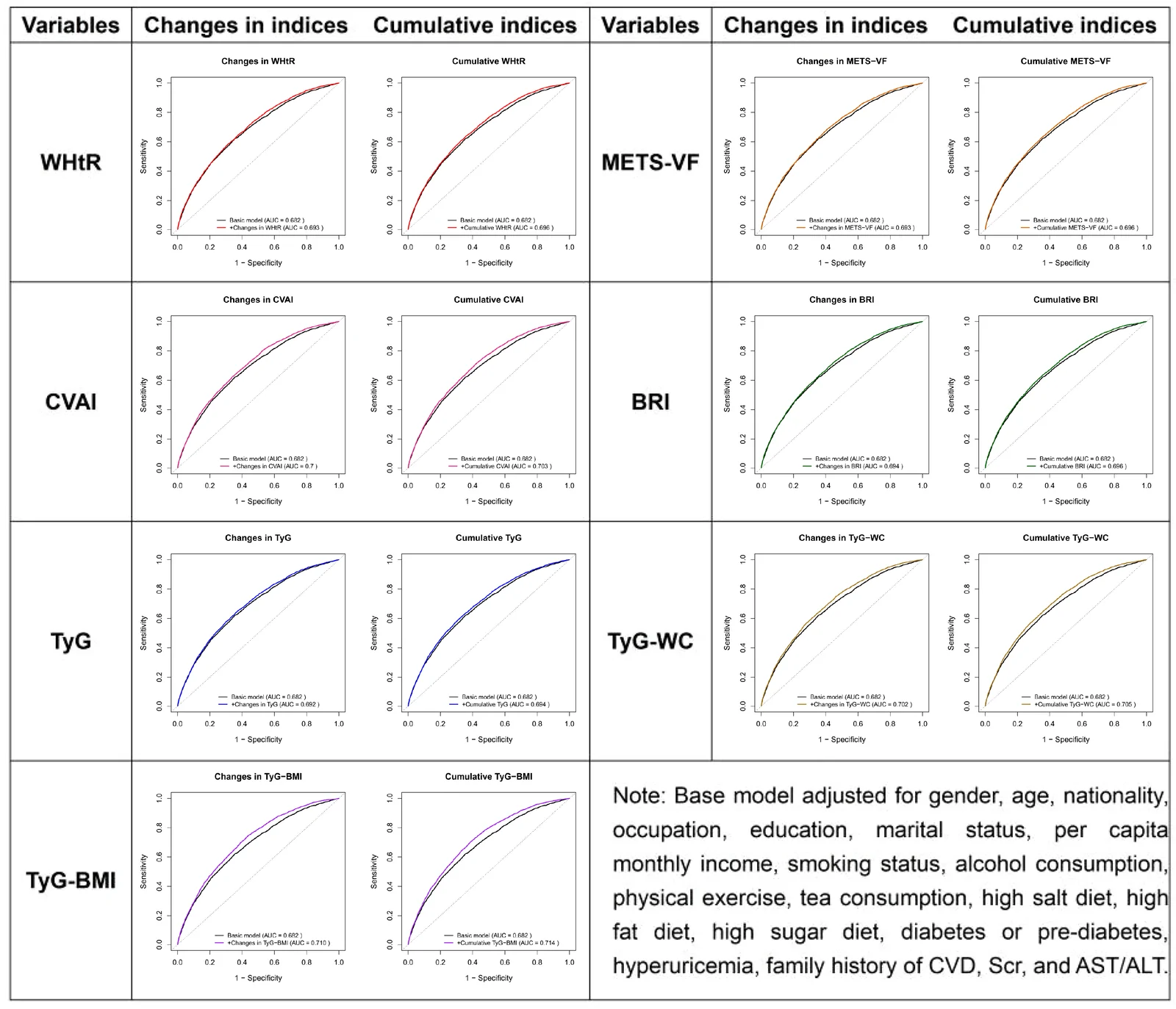
The ROC curves of base model predicting CVD after adding obesity- and lipid-related indices. WHtR, waist-to-height ratio; METS-VF, metabolic score for visceral fat; CVAI, Chinese visceral adiposity index; BRI, body roundness index; TyG, triglyceride-glucose; TyG-WC, TyG-waist circumference; TyG-BMI, TyG-body mass index.

**Table 4:** Improvement in discrimination and risk reclassification for CVD after adding obesity- and lipid-related indices.

Model	AUC (95% CI)	*p*	NRI (95% CI)	*p*	IDI (95% CI)	*p*
Base model^a^	0.682 (0.672 - 0.692)	Ref.	Ref.		Ref.	
+ Changes in WHtR	0.693 (0.683 - 0.703)	<0.001	0.037 (0.021 - 0.054)	<0.001	0.007 (0.006 - 0.009)	<0.001
+ Cumulative WHtR	0.696 (0.687 - 0.706)	<0.001	0.053 (0.036 - 0.070)	<0.001	0.007 (0.005 - 0.008)	<0.001
+ Changes in METS-VF	0.693 (0.684 - 0.703)	<0.001	0.073 (0.056 - 0.091)	<0.001	0.011 (0.009 - 0.013)	<0.001
+ Cumulative METS-VF	0.696 (0.686 - 0.706)	<0.001	0.041 (0.025 - 0.057)	<0.001	0.008 (0.006 - 0.009)	<0.001
+ Changes in CVAI	0.700 (0.691 - 0.710)	<0.001	0.045 (0.030 - 0.060)	<0.001	0.005 (0.004 - 0.007)	<0.001
+ Cumulative CVAI	0.703 (0.693 - 0.713)	<0.001	0.066 (0.048 - 0.085)	<0.001	0.013 (0.011 - 0.015)	<0.001
+ Changes in BRI	0.694 (0.684 - 0.703)	<0.001	0.110 (0.090 - 0.130)	<0.001	0.018 (0.015 - 0.020)	<0.001
+ Cumulative BRI	0.696 (0.686 - 0.706)	<0.001	0.053 (0.036 - 0.069)	<0.001	0.010 (0.008 - 0.011)	<0.001
+ Changes in TyG	0.692 (0.682 - 0.702)	<0.001	0.053 (0.036 - 0.070)	<0.001	0.009 (0.008 - 0.011)	<0.001
+ Cumulative TyG	0.694 (0.685 - 0.704)	<0.001	0.069 (0.051 - 0.087)	<0.001	0.013 (0.011 - 0.015)	<0.001
+ Changes in TyG-WC	0.702 (0.692 - 0.712)	<0.001	0.051 (0.035 - 0.068)	<0.001	0.010 (0.008 - 0.011)	<0.001
+ Cumulative TyG-WC	0.705 (0.695 - 0.715)	<0.001	0.049 (0.034 - 0.064)	<0.001	0.007 (0.006 - 0.009)	<0.001
+Changes in TyG-BMI	0.711 (0.701 - 0.720)	<0.001	0.086 (0.068 - 0.104)	<0.001	0.015 (0.012 - 0.017)	<0.001
+ Cumulative TyG-BMI	0.714 (0.705 - 0.724)	<0.001	0.111 (0.091 - 0.131)	<0.001	0.021 (0.018 - 0.024)	<0.001

WHtR, waist-to-height ratio; METS-VF, metabolic score for visceral fat; CVAI, Chinese visceral adiposity index; BRI, body roundness index; TyG, triglyceride-glucose; TyG-WC, TyG-waist circumference; TyG-BMI, TyG-body mass index.

^a^ Adjusted for gender, age, nationality, occupation, education, marital status, per capita monthly income, smoking status, alcohol consumption, physical exercise, tea consumption, high salt diet, high fat diet, high sugar diet, diabetes or pre-diabetes, hyperuricemia, family history of CVD, Scr, and AST/ALT.

### Subgroup analysis

**Supplementary Figure S7** and **Supplementary Table S4** summarized the subgroup analysis examining the relationships between changes in obesity- and lipid-related indices and incident CVD, while **Supplementary Table S5** summarized the analysis for cumulative values. In most subgroups, the relationship between obesity- and lipid-related indices and new-onset CVD was consistent with the main results after adjusting for potential confounders. Moreover, we also observed that there were interactions between certain subgroups and certain indicators (p for interaction < 0.05).

### Sensitivity analysis

In the sensitivity analysis restricting the outcome to hard cardiovascular events (incident CHD and stroke only), the positive associations between the dynamic changes in obesity- and lipid-related indices and CVD risk remained statistically significant after adjusting for all covariates in Model 3 (**Supplementary Table S6**). Participants in the highest change class of integrated indices consistently presented higher risks of hard CVD compared to the reference group, demonstrating that the observed associations were not solely driven by incident hypertension.

## Discussion

To explore the associations between obesity- and lipid-related indices and CVD, we performed a longitudinal study involving 19,315 participants from the Jinchang Cohort in Northwest China. This large-scale prospective cohort study provides comprehensive evidence on the significant associations between dynamic changes in seven obesity- and lipid-related indices and the risk of incident CVD. The pathogenesis of CVD is often insidious, progressing over years and frequently culminating in catastrophic events such as myocardial infarction or stroke, which severely compromise quality of life and survival. A substantial body of research has sought to identify individuals at high risk, with the majority of existing studies relying solely on single baseline measurements of risk factors (16, 31, 46). Although these baseline indicators provide valuable information, they fail to capture the dynamic nature of metabolic health. Our study addresses this critical gap by demonstrating that longitudinal changes in these indices are strongly associated with CVD risk. This highlights the paramount importance of monitoring temporal trajectories of obesity and lipid parameters—rather than relying on a single assessment—for more accurate risk stratification and timely preventive interventions.

To our knowledge, this is one of the first studies to systematically compare the predictive value of multiple dynamic obesity- and lipid-related indices for CVD in a Chinese population. Leveraging longitudinal data, using K-means clustering model and cumulative calculation as tools for dynamic assessment of obesity- and lipid-related indices change to explore these associations, moving beyond single baseline measurements. Over a median follow-up period of 66 months, the cumulative incidence of CVD was 16.57%. Our findings indicate that participants with the greater increases in WHtR, METS-VF, CVAI, BRI, TyG, TyG-WC, and TyG-BMI had substantially higher risks of developing CVD, even after extensive adjustment for demographic, behavioral, and metabolic confounders. Notably, the indices that integrated lipid and obesity measures—particularly CVAI, TyG-WC, and TyG-BMI—demonstrated the strongest associations, with ORs exceeding 3.5 in the highest change classes. These findings suggest that dynamic changes in these indicators have potential clinical value, allowing for a more comprehensive evaluation of cardiovascular and metabolic changes in participants during follow-up, thereby aiding clinicians in better long-term health management and risk evaluation. The dose-response relationships observed between cumulative exposure to these indices and CVD risk further strengthen the biological plausibility of our findings. In terms of predictive performance, both changes in CVAI and cumulative METS-VF achieved the highest AUC values among all indices. This is consistent with the complex multifactorial nature of CVD, where no single biomarker can fully capture risk. Although the addition of obesity- and lipid-related indices significantly improved the AUC, NRI, and IDI, the absolute magnitude of these improvements was modest, and the discriminative ability of the individual indices was only moderate, with AUC values ranging from approximately 0.60 to 0.66. When added to the baseline model, the AUC increased from 0.682 to approximately 0.692–0.714, indicating that these indices may provide incremental but not definitive predictive value. These findings should therefore be interpreted with caution. The indices may serve as low-cost and easily accessible supplementary markers that provide additional information beyond traditional risk factors, but they are not sufficiently accurate to be used alone for individual risk prediction or clinical decision-making. The statistically significant NRI and IDI indicate improved reclassification at the population level, but statistical significance does not necessarily translate into meaningful clinical benefit. Before these indices can be recommended for routine clinical use, further studies are needed to evaluate their calibration, clinical net benefit using decision curve analysis, cost-effectiveness, and external validity in independent and diverse populations. Future research should also determine whether combining these indices with established risk prediction algorithms improves risk stratification in a clinically meaningful way.

Our findings align with a growing body of evidence highlighting that dynamic changes in adiposity and metabolic health serve as effective predictors of cardiometabolic outcomes (47–53). The superior predictive performance observed for CVAI and TyG-BMI is consistent with their underlying design. CVAI integrates age, BMI, WC, and lipid profiles (38), making it a physiologically and culturally relevant marker of visceral adiposity, particularly in Chinese populations. Data from the China Health and Retirement Longitudinal Study (CHARLS) indicate that individuals with higher baseline CVAI levels or increasing trajectories of CVAI are at elevated risk for cardiometabolic diseases (50). In a population-based cohort of 7,439 participants aged ≥45 years followed for a median of 6.01 years, each standard deviation increase in CVAI was associated with a 17% higher risk of cardiovascular disease (CVD), a 12% increase in heart disease risk, and a 31% increase in stroke risk (54). Another CHARLS analysis of 7,070 participants reported a 57% higher stroke risk per interquartile range increment in CVAI (55). Furthermore, a study of 9,280 individuals from Guizhou Province found that women with high CVAI levels had an increased likelihood of stroke (17). Notably, among the indices evaluated in our study, CVAI is unique in incorporating age—a well-established independent risk factor for cardiovascular disease (56). Accumulating evidence suggests that senescent cells contribute to cardiovascular pathogenesis through the secretion of pro-inflammatory cytokines, chemokines, growth factors, and reactive oxygen species—collectively termed the senescence-associated secretory phenotype (SASP)—which may not strictly correlate with chronological age but warrants consideration in future research (56, 57). Similarly, TyG-BMI combines the TyG index, a surrogate marker of IR, with BMI to reflect both metabolic dysfunction and obesity burden. Our results extend prior cross-sectional and single-timepoint studies by demonstrating that longitudinal deterioration in these indices significantly enhances CVD risk prediction. Previous studies have validated the TyG index and its derivatives as reliable indicators of IR (58–60). For instance, an analysis of the Coronary Artery Risk Development in Young Adults (CARDIA) study, which tracked 4,754 participants over time, showed that a higher long-term trajectory of TyG was associated with increased incident CVD (61). Moreover, cross-sectional evidence indicates that composite indices such as TyG-WC and TyG-BMI outperform TyG alone in identifying insulin resistance (61), a finding corroborated by our results.

The seven indicators selected in this study are well-established surrogates of IR. IR is increasingly recognized as a systemic pathological driver that extends beyond the cardiovascular system (62). A growing body of evidence has linked IR to a broad spectrum of non-communicable diseases, including CVD (62), non-alcoholic fatty liver disease (63), chronic kidney disease (64), and multiple malignancies (65). As a central pathophysiological mechanism connecting obesity and CVD, IR not only disrupts glucose metabolism but also promotes cardiovascular risk through multiple interrelated pathways. These include impaired adipose tissue function, obesity-related inflammation, dyslipidemia, increased production of reactive oxidative species (ROS), atherosclerosis, endothelial dysfunction, and hypertension—all of which contribute to the development and progression of CVD (66, 67). Given its role in elevating the risk of diverse chronic conditions, early and accurate identification of IR using accessible and reliable tools holds significant public health implications. In this context, easily calculable indices serve not only as predictors of CVD risk but also as markers of systemic metabolic dysfunction, offering substantial potential for population-level screening and preventive interventions.

Several limitations should be acknowledged. First, direct measurements of visceral adiposity—such as those obtained via computed tomography (CT), magnetic resonance imaging (MRI), or dual-energy X-ray absorptiometry (DXA)—were not available; therefore, derived metrics such as fat mass index (FMI) and visceral adipose tissue (VAT) could not be included in the analysis. Second, the study population was predominantly composed of Han Chinese individuals from a single occupational cohort, which may limit the generalizability of our findings to other ethnicities or broader populations. Third, although we adjusted for a wide range of potential confounders, residual confounding cannot be completely excluded. Fourth, information on antihypertensive, lipid-lowering, and glucose-lowering medications was not systematically collected, which is an important limitation. These medications can directly affect both the exposure indices and CVD outcomes. Lipid-lowering agents may alter TG, TC, HDL-C, and LDL-C levels and thus influence CVAI, TyG, TyG-WC, TyG-BMI, and METS-VF. Glucose-lowering agents may lower FPG and thereby affect TyG and its derivatives. Antihypertensive agents may lower blood pressure, influence the diagnosis of incident hypertension, and reduce the risk of CHD and stroke. Therefore, unmeasured medication use may have introduced exposure misclassification, residual confounding, and bias in the estimated associations. Although participants with baseline hypertension, CHD, and stroke were excluded, and models were adjusted for diabetes or pre-diabetes, hyperuricemia, Scr, and AST/ALT, these approaches cannot fully capture medication exposure or its metabolic effects. The sensitivity analysis restricted to hard CVD endpoints (incident CHD and stroke only) yielded consistent positive associations, suggesting that the main findings were not solely driven by incident hypertension; however, this analysis cannot completely exclude the influence of medication use. Future studies with detailed longitudinal information on medication class, dose, duration, and adherence are warranted to validate our findings and to examine whether the associations differ by treatment status. Fifth, hypertension was incorporated into our primary composite CVD endpoint alongside CHD and stroke in accordance with ICD-10 circulatory system classifications. While hypertension is an established major precursor and risk factor rather than a traditional hard endpoint (such as MACE), baseline hypertensive individuals were strictly excluded. Nevertheless, this broad definition inevitably contributed to the higher overall incidence rate (16.57%) observed in our cohort. Importantly, our sensitivity analysis restricted strictly to incident CHD and stroke confirmed that dynamic increases in these indices independently predict hard cardiovascular outcomes, underscoring the generalizability and robustness of our conclusions. Sixth, although K-means clustering was used to classify longitudinal change patterns, we did not perform formal stability analyses such as bootstrap resampling with the adjusted Rand index or Jaccard coefficient. K-means assumes roughly spherical clusters and may be sensitive to outliers and the choice of initial centroids. We used the elbow method and Gap statistics to determine the optimal number of clusters, but residual subjectivity in this decision cannot be completely excluded. Future studies should apply alternative clustering methods, such as latent class trajectory models or hierarchical clustering, and formal stability assessments to confirm these change classes. Despite these limitations, this study has several strengths, including a large sample size, a prospective design, extended follow-up duration, and a comprehensive evaluation of both conventional and novel metabolic indices.

## Conclusions

This large-scale longitudinal study demonstrates that dynamic changes in obesity- and lipid-related indices, particularly integrated indices such as CVAI, TyG-BMI, and TyG-WC, are significantly associated with an increased risk of CVD in a Chinese population. Our findings underscore the critical limitation of relying solely on baseline measurements and establish that monitoring the temporal trajectories of these indices provides substantial incremental value for CVD risk prediction beyond traditional models. The application of these easily accessible and low-cost indices as dynamic markers offers a pragmatic adjunctive approach with modest incremental value for enhancing early identification of high-risk individuals, enabling timely interventions, and improving long-term cardiovascular health management, especially in routine clinical practice and resource-constrained settings. Future research should focus on validating these dynamic assessment approaches in diverse populations and exploring their integration into standardized risk prediction algorithms.

## Data Availability

The raw data supporting the conclusions of this article will be made available by the authors, without undue reservation.
