# Supplementary material for: Association between changes in obesity- and lipid-related indices and the risk of cardiovascular disease: a prospective cohort study in Northwest China
Source: Front Endocrinol (Lausanne). 2026 Oct 9;17:1975120. doi: 10.3389/fendo.2026.1975120 (PMC13690539; doi:10.3389/fendo.2026.1975120)
Supplement: Supplementary file 1 [file SupplementaryFile1.docx]

**Table S1.** Definition of WHtR, METS-VF, CVAI, BRI,TyG, TyG-WC, and TyG-BMI.

| **Variables** | **Formulas** |
| --- | --- |
| WHtR | $\text{WHtR = }{\mathrm{WC}_{\mathrm{cm}}}/{{H\mathrm{eight}}_{\mathrm{cm}}}$ |
| METS-VF | $\text{METS-IR = }{\text{Ln ((2 × }\mathrm{FPG}_{\mathrm{md}/dL}\text{ }\text{+ }\mathrm{TG}_{\mathrm{md}/dL}\text{) }\text{× }\mathrm{BMI}_{{kg/m}^{2}}\text{)}}/{\text{Ln (}{HDL-C}_{\mathrm{md}/dL}\text{)}}$  $\begin{matrix} \text{METS-VF }\text{= 4.466 + 0.011(}{\text{Ln (METS-IR)})}^{\text{3}}\text{ }\text{+ 3.239 (}\text{Ln (WHtR))}^{\text{3}}\text{ }\text{+ 0.319 (S}\text{ex}\text{)+ 0.594 (Ln (}{A\mathrm{ge}}_{\mathrm{years}}\text{))} \end{matrix}$ |
| CVAI | Male: CVAI = -267.93 + 0.68 × ${A\mathrm{ge}}_{\mathrm{years}}$ + 0.03 × $\mathrm{BMI}_{{kg/m}^{2}}$ + 4.00 × $\mathrm{WC}_{\mathrm{cm}}$ + 22.00 × Lg ($\mathrm{TG}_{mmol/L}$) - 16.32 ×${HDL-C}_{mmol/L}$  Female: CVAI = -187.32 + 1.71 × ${A\mathrm{ge}}_{\mathrm{years}}$ + 4.23 × $\mathrm{BMI}_{{kg/m}^{2}}$ + 1.12 × $\mathrm{WC}_{\mathrm{cm}}$ + 39.76 × Lg ($\mathrm{TG}_{mmol/L}$) - 11.66 × ${HDL-C}_{mmol/L}$ |
| BRI | $BRI=364.2-365.5\times\sqrt{(1-{{\mathrm{WC}_{\mathrm{cm}}}^{2})}/{(\pi^{2}\times{{H\mathrm{eight}}_{\mathrm{cm}}}^{2})}}$ |
| TyG | $TyG=Ln\left( {\mathrm{TG}_{\mathrm{md}/dL}\times\mathrm{FPG}_{\mathrm{md}/dL}}/2 \right)$ |
| TyG-WC | TyG-WC = TyG × $\mathrm{WC}_{\mathrm{cm}}$ |
| TyG-BMI | TyG-BMI = TyG × $\mathrm{BMI}_{{kg/m}^{2}}$ |

WHtR, waist-to-height ratio; METS-VF, metabolic score for visceral fat; CVAI, Chinese visceral adiposity index; BRI, body roundness index; TyG, triglyceride-glucose; TyG-WC, TyG-waist circumference; TyG-BMI, TyG-body mass index; WC, waist circumference; FBG, fasting blood glucose; TG, triglyceride; HDL-C, high-density lipoprotein cholesterol; BMI,body mass index.

**Table S2.** Associations of cumulative values of obesity- and lipid-related indices with CVD incidence.

|  | Model 1^a^ | | Model 2^b^ | | Model 3^c^ | |
| --- | --- | --- | --- | --- | --- | --- |
|  | OR (95% CI) | P | OR (95% CI) | P | OR (95% CI) | P |
| WHtR | | | | | | |
| Q1 | Ref. |  | Ref. |  | Ref. |  |
| Q2 | 1.343 (1.179 - 1.529) | <0.001 | 1.333 (1.170 - 1.518) | <0.001 | 1.289 (1.130 ~ 1.469) | <0.001 |
| Q3 | 1.832 (1.609 - 2.086) | <0.001 | 1.805 (1.584 - 2.056) | <0.001 | 1.671 (1.464 ~ 1.908) | <0.001 |
| Q4 | 2.609 (2.290 - 2.973) | <0.001 | 2.554 (2.239 - 2.912) | <0.001 | 2.253 (1.969 ~ 2.579) | <0.001 |
| Per SD | 1.448 (1.386 - 1.512) | <0.001 | 1.436 (1.375 - 1.501) | <0.001 | 1.371 (1.311 ~ 1.435) | <0.001 |
| P for trend | <0.001 | | <0.001 | | <0.001 | |
| METS-VF |  | |  | |  | |
| Q1 | Ref. |  | Ref. |  | Ref. |  |
| Q2 | 1.536 (1.340 - 1.762) | <0.001 | 1.562 (1.361 - 1.793) | <0.001 | 1.486 (1.294 ~ 1.707) | <0.001 |
| Q3 | 1.888 (1.651 - 2.159) | <0.001 | 1.903 (1.662 - 2.178) | <0.001 | 1.729 (1.507 ~ 1.984) | <0.001 |
| Q4 | 2.787 (2.437 - 3.187) | <0.001 | 2.788 (2.434 - 3.194) | <0.001 | 2.434 (2.117 ~ 2.799) | <0.001 |
| Per SD | 1.521 (1.447 - 1.599) | <0.001 | 1.513 (1.439 - 1.591) | <0.001 | 1.434 (1.362 ~ 1.511) | <0.001 |
| P for trend | <0.001 | | <0.001 | | <0.001 | |
| CVAI |  | |  | |  | |
| Q1 | Ref. |  | Ref. |  | Ref. |  |
| Q2 | 1.821 (1.582 - 2.096) | <0.001 | 1.819 (1.579 - 2.095) | <0.001 | 1.745 (1.513 ~ 2.013) | <0.001 |
| Q3 | 2.601 (2.245 - 3.014) | <0.001 | 2.579 (2.223 - 2.991) | <0.001 | 2.371 (2.037 ~ 2.759) | <0.001 |
| Q4 | 3.941 (3.377 - 4.600) | <0.001 | 3.869 (3.312 - 4.519) | <0.001 | 3.338 (2.838 ~ 3.925) | <0.001 |
| Per SD | 1.521 (1.447 - 1.599) | <0.001 | 1.513 (1.439 - 1.591) | <0.001 | 1.569 (1.486 ~ 1.658) | <0.001 |
| P for trend | <0.001 | | <0.001 | | <0.001 | |
| BRI |  | |  | |  | |
| Q1 | Ref. |  | Ref. |  | Ref. |  |
| Q2 | 1.323 (1.162 - 1.507) | <0.001 | 1.312 (1.152 - 1.495) | <0.001 | 1.271 (1.115 ~ 1.449) | <0.001 |
| Q3 | 1.838 (1.614 - 2.093) | <0.001 | 1.811 (1.590 - 2.063) | <0.001 | 1.677 (1.470 ~ 1.914) | <0.001 |
| Q4 | 2.587 (2.270 - 2.947) | <0.001 | 2.531 (2.219 - 2.886) | <0.001 | 2.235 (1.953 ~ 2.558) | <0.001 |
| Per SD | 1.428 (1.369 - 1.489) | <0.001 | 1.417 (1.358 - 1.478) | <0.001 | 1.355 (1.296 ~ 1.416) | <0.001 |
| P for trend | <0.001 | | <0.001 | | <0.001 | |
| TyG |  | |  | |  | |
| Q1 | Ref. |  | Ref. |  | Ref. |  |
| Q2 | 1.446 (1.273 - 1.643) | <0.001 | 1.448 (1.274 - 1.645) | <0.001 | 1.396 (1.228 ~ 1.588) | <0.001 |
| Q3 | 1.762 (1.555 - 1.997) | <0.001 | 1.765 (1.557 - 2.002) | <0.001 | 1.622 (1.427 ~ 1.842) | <0.001 |
| Q4 | 2.497 (2.209 - 2.824) | <0.001 | 2.492 (2.201 - 2.821) | <0.001 | 2.112 (1.853 ~ 2.406) | <0.001 |
| Per SD | 1.428 (1.369 - 1.489) | <0.001 | 1.417 (1.358 - 1.478) | <0.001 | 1.310 (1.254 ~ 1.368) | <0.001 |
| P for trend | <0.001 | | <0.001 | | <0.001 | |
| TyG-WC |  | |  | |  | |
| Q1 | Ref. |  | Ref. |  | Ref. |  |
| Q2 | 1.714 (1.503 - 1.956) | <0.001 | 1.701 (1.490 - 1.941) | <0.001 | 1.641 (1.437 ~ 1.875) | <0.001 |
| Q3 | 2.291 (1.989 - 2.638) | <0.001 | 2.262 (1.963 - 2.606) | <0.001 | 2.084 (1.803 ~ 2.408) | <0.001 |
| Q4 | 3.885 (3.356 - 4.497) | <0.001 | 3.822 (3.299 - 4.428) | <0.001 | 3.257 (2.789 ~ 3.805) | <0.001 |
| Per SD | 1.673 (1.594 - 1.756) | <0.001 | 1.666 (1.587 - 1.750) | <0.001 | 1.577 (1.496 ~ 1.662) | <0.001 |
| P for trend | <0.001 | | <0.001 | | <0.001 | |
| TyG-BMI |  | |  | |  | |
| Q1 | Ref. |  | Ref. |  | Ref. |  |
| Q2 | 1.584 (1.384 - 1.812) | <0.001 | 1.585 (1.384 - 1.814) | <0.001 | 1.546 (1.349 ~ 1.771) | <0.001 |
| Q3 | 2.206 (1.935 - 2.515) | <0.001 | 2.199 (1.928 - 2.509) | <0.001 | 2.079 (1.818 ~ 2.379) | <0.001 |
| Q4 | 3.639 (3.201 - 4.138) | <0.001 | 3.620 (3.181 - 4.120) | <0.001 | 3.239 (2.825 ~ 3.713) | <0.001 |
| Per SD | 1.655 (1.588 - 1.725) | <0.001 | 1.651 (1.584 - 1.722) | <0.001 | 1.596 (1.526 ~ 1.670) | <0.001 |
| P for trend | <0.001 | | <0.001 | | <0.001 | |

WHtR,waist-to-height ratio; METS-VF, metabolic score for visceral fat; CVAI, Chinese visceral adiposity index; BRI, body roundness index; TyG, triglyceride-glucose; TyG-WC, TyG-waist circumference; TyG-BMI, TyG-body mass index; OR, odds ratio; CI, confidence interval.

^a^ Adjusted for gender and age;

^b^Adjusted for gender, age, nationality, occupation, education, marital status, per capita monthly income, smoking status, alcohol consumption, physical exercise, tea consumption, high salt diet, high fat diet, and high sugar diet.

^c^Adjusted for variables in Model 2 and diabetes or pre-diabetes, hyperuricemia, family history of CVD, Scr, and AST/ALT.

Figure S1. Clustering of the changes in METS-VF from baseline to follow-up. (A-B) Determine the optimal number of clusters for the changes in METS-VF using the Within-Cluster Sum of Squares (WSS) and Gap Statistics methods; (C) Changes in METS-VF classified into three classes using the K-means algorithm; (D) Data visualization of mean METS-VF across different classes; (E-F) Distribution of METS-VF at baseline or follow-up.

Figure S2. Clustering of the changes in CVAI from baseline to follow-up. (A-B) Determine the optimal number of clusters for the changes in CVAI using the Within-Cluster Sum of Squares (WSS) and Gap Statistics methods; (C) Changes in CVAI classified into four classes using the K-means algorithm; (D) Data visualization of mean CVAI across different classes; (E-F) Distribution of CVAI at baseline or follow-up.

Figure S3. Clustering of the changes in BRI from baseline to follow-up. (A-B) Determine the optimal number of clusters for the changes in BRI using the Within-Cluster Sum of Squares (WSS) and Gap Statistics methods; (C) Changes in BRI classified into three classes using the K-means algorithm; (D) Data visualization of mean BRI across different classes; (E-F) Distribution of BRI at baseline or follow-up.

Figure S4. Clustering of the changes in TyG from baseline to follow-up. (A-B) Determine the optimal number of clusters for the changes in TyG using the Within-Cluster Sum of Squares (WSS) and Gap Statistics methods; (C) Changes in TyG classified into three classes using the K-means algorithm; (D) Data visualization of mean TyG across different classes; (E-F) Distribution of TyG at baseline or follow-up.

Figure S5. Clustering of the changes in TyG-WC from baseline to follow-up. (A-B) Determine the optimal number of clusters for the changes in TyG-WC using the Within-Cluster Sum of Squares (WSS) and Gap Statistics methods; (C) Changes in TyG-WC classified into four classes using the K-means algorithm; (D) Data visualization of mean TyG-WC across different classes; (E-F) Distribution of TyG-WC at baseline or follow-up.

Figure S6. Clustering of the changes in TyG-BMI from baseline to follow-up. (A-B) Determine the optimal number of clusters for the changes in TyG-BMI using the Within-Cluster Sum of Squares (WSS) and Gap Statistics methods; (C) Changes in TyG-BMI classified into four classes using the K-means algorithm; (D) Data visualization of mean TyG-BMI across different classes; (E-F) Distribution of TyG-BMI at baseline or follow-up.

Table S3. Subgroup and interaction analyses among changes in WHtR, METS-VF, BRI, TyG (3 classes) and CVD across various subgroups. Adjusted for gender, age, nationality, occupation, education, marital status, per capita monthly income, smoking status, alcohol consumption, physical exercise, tea consumption, high salt diet, high fat diet, high sugar diet, diabetes or pre-diabetes, hyperuricemia, family history of CVD, Scr, and AST/ALT., except in the corresponding group.

| Subgroup | N | Class | WHTR | | METS-VF | | BRI | | TyG | |
| --- | --- | --- | --- | --- | --- | --- | --- | --- | --- | --- |
|  |  |  | OR (95% CI) | P for interaction | OR (95% CI) | P for interaction | OR (95% CI) | P for interaction | OR (95% CI) | P for interaction |
| Sex |  |  |  | 0.893 |  | 0.304 |  | 0.975 |  | 0.232 |
| Male | 11292 | Class2 | 1.525 (1.295-1.797) |  | 2.047 (1.657-2.528) |  | 1.559 (1.338-1.817) |  | 1.493 (1.308-1.703) |  |
|  |  | Class3 | 2.345 (1.977-2.781) |  | 3.334 (2.686-4.138) |  | 2.408 (2.045-2.834) |  | 2.034 (1.746-2.371) |  |
| Female | 8023 | Class2 | 1.5 (1.305-1.725) |  | 1.467 (1.197-1.797) |  | 1.492 (1.297-1.716) |  | 1.633 (1.419-1.88) |  |
|  |  | Class3 | 2.338 (1.899-2.878) |  | 2.45 (1.981-3.03) |  | 2.315 (1.862-2.879) |  | 1.809 (1.441-2.27) |  |
| Age |  |  |  | 0.341 |  | 0.203 |  | 0.128 |  | 0.013 |
| >= 45 years | 6889 | Class2 | 1.373 (1.177-1.602) |  | 1.389 (1.036-1.863) |  | 1.345 (1.161-1.558) |  | 1.48 (1.287-1.702) |  |
|  |  | Class3 | 2.055 (1.727-2.446) |  | 2.26 (1.697-3.009) |  | 2.007 (1.692-2.382) |  | 1.698 (1.427-2.021) |  |
| < 45 years | 12426 | Class2 | 1.585 (1.373-1.83) |  | 1.772 (1.496-2.099) |  | 1.626 (1.412-1.873) |  | 1.603 (1.403-1.832) |  |
|  |  | Class3 | 2.431 (2.051-2.881) |  | 2.856 (2.38-3.427) |  | 2.543 (2.144-3.016) |  | 2.314 (1.943-2.755) |  |
| Nationality |  |  |  | 0.479 |  | 0.476 |  | 0.245 |  | 0.822 |
| Han nationality | 18716 | Class2 | 1.488 (1.339-1.654) |  | 1.737 (1.499-2.013) |  | 1.495 (1.349-1.656) |  | 1.54 (1.398-1.697) |  |
|  |  | Class3 | 2.255 (1.996-2.549) |  | 2.82 (2.422-3.283) |  | 2.267 (2.007-2.561) |  | 1.972 (1.742-2.232) |  |
| Others | 599 | Class2 | 2.151 (1.112-4.161) |  | 1.245 (0.553-2.803) |  | 1.994 (1.053-3.777) |  | 1.764 (0.975-3.19) |  |
|  |  | Class3 | 4.229 (1.983-9.023) |  | 2.703 (1.173-6.23) |  | 5.132 (2.425-10.861) |  | 2.177 (1.01-4.693) |  |
| Occupation |  |  |  | 0.106 |  | 0.967 |  | 0.045 |  | 0.326 |
| Woker | 15825 | Class2 | 1.627 (1.449-1.828) |  | 1.772 (1.51-2.079) |  | 1.645 (1.47-1.841) |  | 1.57 (1.413-1.745) |  |
|  |  | Class3 | 2.439 (2.133-2.79) |  | 2.984 (2.531-3.517) |  | 2.479 (2.169-2.833) |  | 2.11 (1.843-2.414) |  |
| Others | 3490 | Class2 | 1.038 (0.814-1.324) |  | 1.529 (1.076-2.172) |  | 0.983 (0.773-1.249) |  | 1.407 (1.123-1.763) |  |
|  |  | Class3 | 1.732 (1.307-2.295) |  | 2.127 (1.481-3.055) |  | 1.683 (1.269-2.231) |  | 1.466 (1.091-1.971) |  |
| Education |  |  |  | 0.058 |  | 0.18 |  | 0.038 |  | 0.079 |
| Middle school or below | 5667 | Class2 | 1.476 (1.24-1.756) |  | 1.521 (1.136-2.037) |  | 1.504 (1.273-1.778) |  | 1.45 (1.242-1.693) |  |
|  |  | Class3 | 2.307 (1.895-2.809) |  | 2.584 (1.937-3.448) |  | 2.349 (1.934-2.852) |  | 1.764 (1.448-2.149) |  |
| High school or equality | 5909 | Class2 | 1.402 (1.166-1.684) |  | 1.509 (1.17-1.946) |  | 1.405 (1.175-1.682) |  | 1.522 (1.278-1.813) |  |
|  |  | Class3 | 2.096 (1.683-2.611) |  | 2.328 (1.793-3.023) |  | 2.046 (1.639-2.554) |  | 2.025 (1.621-2.53) |  |
| College or above | 7739 | Class2 | 1.582 (1.31-1.91) |  | 1.935 (1.547-2.421) |  | 1.557 (1.296-1.872) |  | 1.646 (1.387-1.954) |  |
|  |  | Class3 | 2.339 (1.882-2.907) |  | 3.102 (2.434-3.952) |  | 2.409 (1.941-2.99) |  | 2.138 (1.712-2.67) |  |
| Marital status |  |  |  | 0.043 |  | 0.074 |  | 0.058 |  | 0.684 |
| Single | 2208 | Class2 | 1.689 (1.18-2.418) |  | 2.085 (1.444-3.011) |  | 1.836 (1.297-2.598) |  | 1.609 (1.173-2.207) |  |
|  |  | Class3 | 3.224 (2.171-4.787) |  | 4.248 (2.715-6.646) |  | 3.037 (2.03-4.545) |  | 2.097 (1.357-3.238) |  |
| Married | 17107 | Class2 | 1.475 (1.322-1.645) |  | 1.628 (1.39-1.907) |  | 1.467 (1.319-1.632) |  | 1.531 (1.385-1.693) |  |
|  |  | Class3 | 2.206 (1.943-2.506) |  | 2.639 (2.246-3.101) |  | 2.246 (1.979-2.549) |  | 1.959 (1.724-2.226) |  |
| Per capita monthly income |  |  |  | 0.791 |  | 0.168 |  | 0.986 |  | 0.527 |
| < 2000 yuan | 9946 | Class2 | 1.479 (1.277-1.712) |  | 1.55 (1.264-1.9) |  | 1.538 (1.334-1.773) |  | 1.574 (1.378-1.798) |  |
|  |  | Class3 | 2.287 (1.934-2.704) |  | 2.719 (2.21-3.345) |  | 2.401 (2.033-2.835) |  | 2.16 (1.826-2.554) |  |
| >= 2000yuan | 9369 | Class2 | 1.514 (1.304-1.757) |  | 1.894 (1.54-2.329) |  | 1.455 (1.259-1.681) |  | 1.516 (1.321-1.74) |  |
|  |  | Class3 | 2.277 (1.912-2.712) |  | 2.86 (2.305-3.549) |  | 2.203 (1.85-2.623) |  | 1.786 (1.492-2.138) |  |
| Smoking status |  |  |  | 0.779 |  | 0.368 |  | 0.847 |  | 0.076 |
| Not smoking or quitting smoking | 12068 | Class2 | 1.474 (1.304-1.667) |  | 1.599 (1.342-1.904) |  | 1.479 (1.311-1.67) |  | 1.541 (1.374-1.729) |  |
|  |  | Class3 | 2.311 (1.983-2.693) |  | 2.666 (2.223-3.198) |  | 2.326 (1.992-2.716) |  | 1.781 (1.514-2.096) |  |
| Yes | 7247 | Class2 | 1.563 (1.276-1.916) |  | 1.996 (1.534-2.598) |  | 1.559 (1.29-1.884) |  | 1.585 (1.33-1.889) |  |
|  |  | Class3 | 2.317 (1.873-2.866) |  | 3.162 (2.419-4.134) |  | 2.337 (1.908-2.863) |  | 2.227 (1.826-2.715) |  |
| Alcohol consumption |  |  |  | 0.709 |  | 0.081 |  | 0.46 |  | 0.081 |
| Not drinking or quitting drinking | 15436 | Class2 | 1.526 (1.362-1.71) |  | 1.709 (1.454-2.009) |  | 1.532 (1.371-1.712) |  | 1.61 (1.449-1.789) |  |
|  |  | Class3 | 2.366 (2.066-2.71) |  | 2.946 (2.495-3.479) |  | 2.431 (2.122-2.785) |  | 1.942 (1.685-2.239) |  |
| Yes | 3879 | Class2 | 1.338 (1.027-1.744) |  | 1.71 (1.225-2.388) |  | 1.331 (1.039-1.705) |  | 1.291 (1.028-1.623) |  |
|  |  | Class3 | 1.961 (1.484-2.59) |  | 2.327 (1.648-3.284) |  | 1.9 (1.454-2.482) |  | 1.894 (1.468-2.445) |  |
| Physical exercise |  |  |  | 0.053 |  | 0.834 |  | 0.165 |  | 0.364 |
| Never | 2896 | Class2 | 2.06 (1.52-2.791) |  | 1.658 (1.139-2.414) |  | 1.864 (1.398-2.484) |  | 1.597 (1.223-2.086) |  |
|  |  | Class3 | 3.327 (2.367-4.676) |  | 3.051 (2.071-4.495) |  | 2.816 (2.023-3.92) |  | 2.208 (1.586-3.074) |  |
| Occasionally | 8540 | Class2 | 1.49 (1.269-1.75) |  | 1.565 (1.271-1.925) |  | 1.5 (1.283-1.754) |  | 1.671 (1.439-1.941) |  |
|  |  | Class3 | 2.094 (1.739-2.522) |  | 2.585 (2.084-3.207) |  | 2.101 (1.745-2.531) |  | 1.991 (1.641-2.417) |  |
| Regularly | 7879 | Class2 | 1.398 (1.197-1.633) |  | 1.942 (1.522-2.479) |  | 1.43 (1.229-1.664) |  | 1.438 (1.249-1.656) |  |
|  |  | Class3 | 2.24 (1.867-2.686) |  | 3.037 (2.368-3.896) |  | 2.385 (1.99-2.859) |  | 1.903 (1.587-2.281) |  |
| Tea consumption |  |  |  | 0.677 |  | 0.386 |  | 0.659 |  | 0.038 |
| Never | 9361 | Class2 | 1.422 (1.237-1.636) |  | 1.771 (1.454-2.157) |  | 1.412 (1.231-1.621) |  | 1.678 (1.466-1.921) |  |
|  |  | Class3 | 2.247 (1.887-2.676) |  | 2.848 (2.319-3.497) |  | 2.27 (1.903-2.709) |  | 1.897 (1.57-2.293) |  |
| Occasionally or regularly | 9954 | Class2 | 1.584 (1.351-1.858) |  | 1.619 (1.305-2.007) |  | 1.601 (1.375-1.864) |  | 1.414 (1.235-1.619) |  |
|  |  | Class3 | 2.391 (2.01-2.844) |  | 2.703 (2.171-3.366) |  | 2.422 (2.043-2.871) |  | 1.981 (1.684-2.33) |  |
| High salt diet |  |  |  | 0.567 |  | 0.83 |  | 0.855 |  | 0.683 |
| No | 15166 | Class2 | 1.515 (1.347-1.704) |  | 1.725 (1.467-2.028) |  | 1.499 (1.337-1.68) |  | 1.561 (1.4-1.74) |  |
|  |  | Class3 | 2.224 (1.937-2.554) |  | 2.778 (2.348-3.285) |  | 2.247 (1.956-2.58) |  | 1.981 (1.721-2.28) |  |
| Yes | 4149 | Class2 | 1.43 (1.136-1.8) |  | 1.672 (1.203-2.325) |  | 1.508 (1.208-1.882) |  | 1.493 (1.22-1.827) |  |
|  |  | Class3 | 2.448 (1.904-3.148) |  | 2.883 (2.068-4.02) |  | 2.502 (1.953-3.206) |  | 1.94 (1.51-2.492) |  |
| High fat diet |  |  |  | 0.532 |  | 0.668 |  | 0.492 |  | 0.403 |
| No | 15750 | Class2 | 1.516 (1.351-1.701) |  | 1.761 (1.503-2.064) |  | 1.513 (1.353-1.693) |  | 1.577 (1.418-1.755) |  |
|  |  | Class3 | 2.246 (1.961-2.573) |  | 2.833 (2.403-3.341) |  | 2.245 (1.96-2.571) |  | 1.953 (1.701-2.243) |  |
| Yes | 3565 | Class2 | 1.424 (1.113-1.821) |  | 1.523 (1.063-2.183) |  | 1.445 (1.139-1.834) |  | 1.448 (1.165-1.799) |  |
|  |  | Class3 | 2.442 (1.869-3.192) |  | 2.7 (1.885-3.866) |  | 2.54 (1.951-3.308) |  | 2.108 (1.614-2.751) |  |
| High sugar diet |  |  |  | 0.03 |  | 0.037 |  | 0.07 |  | 0.194 |
| No | 15480 | Class2 | 1.412 (1.256-1.589) |  | 1.597 (1.362-1.872) |  | 1.419 (1.265-1.591) |  | 1.473 (1.321-1.643) |  |
|  |  | Class3 | 2.103 (1.837-2.409) |  | 2.542 (2.157-2.996) |  | 2.18 (1.905-2.495) |  | 1.899 (1.655-2.179) |  |
| Yes | 3835 | Class2 | 1.903 (1.514-2.391) |  | 2.491 (1.736-3.574) |  | 1.88 (1.51-2.34) |  | 1.832 (1.503-2.234) |  |
|  |  | Class3 | 3.131 (2.399-4.087) |  | 4.449 (3.08-6.428) |  | 2.856 (2.191-3.723) |  | 2.291 (1.744-3.009) |  |
| Diabetes or pre−diabetes |  |  |  | <0.001 |  | 0.002 |  | <0.001 |  | 0.221 |
| No | 16209 | Class2 | 1.636 (1.456-1.838) |  | 1.811 (1.545-2.122) |  | 1.64 (1.465-1.837) |  | 1.556 (1.404-1.725) |  |
|  |  | Class3 | 2.616 (2.282-2.999) |  | 3.092 (2.624-3.643) |  | 2.637 (2.3-3.022) |  | 2.069 (1.796-2.383) |  |
| Yes | 3106 | Class2 | 0.982 (0.773-1.248) |  | 1.139 (0.786-1.649) |  | 0.992 (0.789-1.248) |  | 1.439 (1.103-1.878) |  |
|  |  | Class3 | 1.345 (1.037-1.744) |  | 1.634 (1.131-2.36) |  | 1.377 (1.066-1.778) |  | 1.735 (1.314-2.291) |  |
| Hyperuricemia |  |  |  | 0.141 |  | 0.264 |  | 0.096 |  | 0.044 |
| No | 17451 | Class2 | 1.489 (1.337-1.659) |  | 1.684 (1.448-1.958) |  | 1.505 (1.355-1.672) |  | 1.555 (1.409-1.717) |  |
|  |  | Class3 | 2.35 (2.07-2.669) |  | 2.793 (2.391-3.262) |  | 2.392 (2.106-2.717) |  | 1.845 (1.616-2.106) |  |
| Yes | 1864 | Class2 | 1.429 (0.919-2.22) |  | 2.049 (1.162-3.61) |  | 1.26 (0.844-1.883) |  | 1.493 (1.021-2.184) |  |
|  |  | Class3 | 1.892 (1.212-2.955) |  | 3.05 (1.723-5.399) |  | 1.727 (1.147-2.601) |  | 2.638 (1.783-3.904) |  |
| Family history of CVD |  |  |  | 0.702 |  | 0.577 |  | 0.644 |  | 0.65 |
| No | 12130 | Class2 | 1.479 (1.291-1.695) |  | 1.799 (1.489-2.174) |  | 1.482 (1.3-1.689) |  | 1.516 (1.343-1.712) |  |
|  |  | Class3 | 2.31 (1.981-2.693) |  | 2.879 (2.369-3.497) |  | 2.337 (2.007-2.721) |  | 1.87 (1.6-2.185) |  |
| Yes | 7185 | Class2 | 1.558 (1.323-1.834) |  | 1.626 (1.296-2.04) |  | 1.568 (1.336-1.842) |  | 1.611 (1.38-1.882) |  |
|  |  | Class3 | 2.232 (1.833-2.72) |  | 2.706 (2.141-3.419) |  | 2.232 (1.831-2.721) |  | 2.177 (1.785-2.656) |  |

Table S4. Subgroup and interaction analyses among changes in CVAI, TyG-WC, TyG-BMI (4 classes) and CVD across various subgroups. Adjusted for gender, age, nationality, occupation, education, marital status, per capita monthly income, smoking status, alcohol consumption, physical exercise, tea consumption, high salt diet, high fat diet, high sugar diet, diabetes or pre-diabetes, hyperuricemia, family history of CVD, Scr, and AST/ALT., except in the corresponding group.

| Subgroup | N | Class | CVAI | | TyG-WC | | TyG-BMI | |
| --- | --- | --- | --- | --- | --- | --- | --- | --- |
|  |  |  | OR (95% CI) | P for interaction | OR (95% CI) | P for interaction | OR (95% CI) | P for interaction |
| Sex |  |  |  | 0.383 |  | 0.888 |  | 0.454 |
| Male | 11292 | Class2 | 1.443 (1.044-1.994) |  | 1.826 (1.36-2.451) |  | 1.707 (1.4-2.08) |  |
|  |  | Class3 | 2.509 (1.835-3.432) |  | 2.744 (2.054-3.666) |  | 2.63 (2.161-3.2) |  |
|  |  | Class4 | 3.844 (2.794-5.288) |  | 4.333 (3.213-5.842) |  | 4.178 (3.373-5.176) |  |
| Female | 8023 | Class2 | 2.034 (1.709-2.421) |  | 1.716 (1.484-1.985) |  | 1.604 (1.354-1.901) |  |
|  |  | Class3 | 3.72 (2.996-4.62) |  | 2.655 (2.165-3.256) |  | 2.529 (2.097-3.05) |  |
|  |  | Class4 | 5.724 (4.047-8.097) |  | 3.713 (2.408-5.725) |  | 4.567 (3.536-5.9) |  |
| Age |  |  |  | 0.082 |  | 0.157 |  | <0.001 |
| >= 45 years | 6889 | Class2 | 1.368 (0.999-1.873) |  | 1.712 (1.414-2.074) |  | 1.41 (1.17-1.699) |  |
|  |  | Class3 | 2.407 (1.761-3.289) |  | 2.337 (1.894-2.883) |  | 1.886 (1.559-2.282) |  |
|  |  | Class4 | 3.322 (2.395-4.607) |  | 3.277 (2.568-4.181) |  | 2.788 (2.229-3.486) |  |
| < 45 years | 12426 | Class2 | 2.143 (1.798-2.553) |  | 1.766 (1.488-2.095) |  | 1.867 (1.563-2.23) |  |
|  |  | Class3 | 3.568 (2.888-4.409) |  | 2.943 (2.405-3.601) |  | 3.356 (2.791-4.035) |  |
|  |  | Class4 | 5.932 (4.659-7.553) |  | 4.829 (3.837-6.077) |  | 5.741 (4.643-7.098) |  |
| Nationality |  |  |  | 0.343 |  | 0.216 |  | 0.829 |
| Han nationality | 18716 | Class2 | 1.842 (1.584-2.141) |  | 1.773 (1.56-2.015) |  | 1.657 (1.456-1.886) |  |
|  |  | Class3 | 3.055 (2.599-3.591) |  | 2.589 (2.24-2.994) |  | 2.524 (2.209-2.884) |  |
|  |  | Class4 | 4.61 (3.85-5.52) |  | 3.934 (3.327-4.653) |  | 4.045 (3.465-4.723) |  |
| Others | 599 | Class2 | 1.188 (0.493-2.863) |  | 1.165 (0.53-2.563) |  | 1.599 (0.733-3.489) |  |
|  |  | Class3 | 3.167 (1.217-8.239) |  | 3.389 (1.401-8.198) |  | 3.337 (1.506-7.393) |  |
|  |  | Class4 | 6.892 (2.351-20.2) |  | 7.049 (2.554-19.459) |  | 6.285 (2.437-16.207) |  |
| Occupation |  |  |  | 0.462 |  | 0.216 |  | 0.05 |
| Woker | 15825 | Class2 | 1.912 (1.622-2.253) |  | 1.91 (1.659-2.199) |  | 1.837 (1.593-2.118) |  |
|  |  | Class3 | 3.264 (2.736-3.893) |  | 2.798 (2.386-3.281) |  | 2.776 (2.395-3.216) |  |
|  |  | Class4 | 4.813 (3.953-5.86) |  | 4.268 (3.552-5.127) |  | 4.631 (3.908-5.487) |  |
| Others | 3490 | Class2 | 1.428 (1.009-2.023) |  | 1.207 (0.906-1.608) |  | 1.067 (0.797-1.427) |  |
|  |  | Class3 | 2.162 (1.481-3.155) |  | 1.85 (1.326-2.581) |  | 1.701 (1.264-2.289) |  |
|  |  | Class4 | 3.754 (2.467-5.712) |  | 2.997 (2.031-4.422) |  | 2.332 (1.628-3.34) |  |
| Education |  |  |  | 0.02 |  | <0.001 |  | 0.003 |
| Middle school or below | 5667 | Class2 | 1.664 (1.252-2.211) |  | 2.022 (1.64-2.494) |  | 1.739 (1.407-2.148) |  |
|  |  | Class3 | 2.754 (2.055-3.692) |  | 2.54 (2.011-3.21) |  | 2.347 (1.889-2.917) |  |
|  |  | Class4 | 4.291 (3.128-5.887) |  | 3.917 (2.981-5.148) |  | 3.683 (2.855-4.751) |  |
| High school or equality | 5909 | Class2 | 1.481 (1.146-1.914) |  | 1.401 (1.125-1.746) |  | 1.359 (1.088-1.697) |  |
|  |  | Class3 | 2.503 (1.896-3.305) |  | 2.239 (1.74-2.881) |  | 2.276 (1.808-2.866) |  |
|  |  | Class4 | 3.416 (2.492-4.684) |  | 3.381 (2.509-4.557) |  | 3.535 (2.696-4.635) |  |
| College or above | 7739 | Class2 | 2.206 (1.729-2.815) |  | 1.831 (1.452-2.31) |  | 1.902 (1.503-2.406) |  |
|  |  | Class3 | 3.803 (2.87-5.039) |  | 3.131 (2.399-4.087) |  | 3.171 (2.489-4.041) |  |
|  |  | Class4 | 6.032 (4.399-8.272) |  | 4.715 (3.5-6.352) |  | 5.221 (3.962-6.881) |  |
| Marital status |  |  |  | 0.157 |  | 0.142 |  | 0.42 |
| Single | 2208 | Class2 | 2.096 (1.286-3.417) |  | 1.954 (1.222-3.125) |  | 1.468 (0.962-2.241) |  |
|  |  | Class3 | 2.931 (1.751-4.907) |  | 3.551 (2.144-5.882) |  | 2.711 (1.755-4.187) |  |
|  |  | Class4 | 5.852 (3.322-10.308) |  | 6.143 (3.499-10.784) |  | 5.323 (3.258-8.698) |  |
| Married | 17107 | Class2 | 1.738 (1.486-2.032) |  | 1.721 (1.51-1.963) |  | 1.655 (1.447-1.892) |  |
|  |  | Class3 | 2.967 (2.508-3.511) |  | 2.523 (2.172-2.93) |  | 2.491 (2.169-2.86) |  |
|  |  | Class4 | 4.417 (3.661-5.329) |  | 3.812 (3.204-4.535) |  | 3.949 (3.362-4.638) |  |
| Per capita monthly income |  |  |  | 0.998 |  | 0.427 |  | 0.485 |
| < 2000 yuan | 9946 | Class2 | 1.891 (1.535-2.329) |  | 1.774 (1.481-2.126) |  | 1.751 (1.463-2.096) |  |
|  |  | Class3 | 3.246 (2.595-4.062) |  | 2.903 (2.375-3.549) |  | 2.866 (2.384-3.444) |  |
|  |  | Class4 | 4.959 (3.873-6.351) |  | 4.183 (3.322-5.268) |  | 4.38 (3.54-5.418) |  |
| >= 2000yuan | 9369 | Class2 | 1.731 (1.401-2.139) |  | 1.716 (1.439-2.047) |  | 1.557 (1.299-1.866) |  |
|  |  | Class3 | 2.851 (2.27-3.58) |  | 2.291 (1.867-2.812) |  | 2.21 (1.829-2.669) |  |
|  |  | Class4 | 4.307 (3.332-5.568) |  | 3.763 (2.965-4.777) |  | 3.762 (3.019-4.689) |  |
| Smoking status |  |  |  | 0.526 |  | 0.611 |  | 0.978 |
| Not smoking or quitting smoking | 12068 | Class2 | 1.936 (1.648-2.274) |  | 1.752 (1.53-2.007) |  | 1.644 (1.416-1.91) |  |
|  |  | Class3 | 3.228 (2.687-3.877) |  | 2.533 (2.144-2.993) |  | 2.564 (2.189-3.003) |  |
|  |  | Class4 | 4.893 (3.92-6.106) |  | 3.688 (2.971-4.577) |  | 4.061 (3.346-4.93) |  |
| Yes | 7247 | Class2 | 1.383 (0.917-2.086) |  | 1.827 (1.25-2.671) |  | 1.668 (1.304-2.134) |  |
|  |  | Class3 | 2.491 (1.674-3.708) |  | 2.847 (1.961-4.135) |  | 2.525 (1.978-3.222) |  |
|  |  | Class4 | 3.776 (2.517-5.664) |  | 4.501 (3.065-6.608) |  | 4.155 (3.184-5.421) |  |
| Alcohol consumption |  |  |  | 0.392 |  | 0.952 |  | 0.857 |
| Not drinking or quitting drinking | 15436 | Class2 | 1.877 (1.604-2.196) |  | 1.739 (1.525-1.983) |  | 1.652 (1.439-1.897) |  |
|  |  | Class3 | 3.302 (2.781-3.92) |  | 2.628 (2.254-3.063) |  | 2.594 (2.247-2.995) |  |
|  |  | Class4 | 5.061 (4.153-6.167) |  | 4.094 (3.4-4.93) |  | 4.263 (3.592-5.059) |  |
| Yes | 3879 | Class2 | 1.27 (0.797-2.026) |  | 1.834 (1.109-3.034) |  | 1.626 (1.162-2.275) |  |
|  |  | Class3 | 1.841 (1.16-2.923) |  | 2.561 (1.547-4.242) |  | 2.302 (1.644-3.223) |  |
|  |  | Class4 | 2.813 (1.75-4.521) |  | 3.804 (2.265-6.387) |  | 3.489 (2.423-5.024) |  |
| Physical exercise |  |  |  | 0.154 |  | 0.268 |  | 0.712 |
| Never | 2896 | Class2 | 1.785 (1.174-2.715) |  | 2.206 (1.507-3.229) |  | 1.566 (1.109-2.211) |  |
|  |  | Class3 | 3.716 (2.405-5.742) |  | 3.786 (2.5-5.732) |  | 2.332 (1.639-3.319) |  |
|  |  | Class4 | 5.608 (3.466-9.074) |  | 5.753 (3.627-9.124) |  | 4.213 (2.835-6.262) |  |
| Occasionally | 8540 | Class2 | 1.899 (1.531-2.355) |  | 1.775 (1.462-2.154) |  | 1.827 (1.497-2.229) |  |
|  |  | Class3 | 3.218 (2.535-4.087) |  | 2.577 (2.065-3.216) |  | 2.811 (2.29-3.45) |  |
|  |  | Class4 | 4.329 (3.3-5.677) |  | 3.747 (2.901-4.839) |  | 4.4 (3.466-5.586) |  |
| Regularly | 7879 | Class2 | 1.74 (1.373-2.205) |  | 1.644 (1.365-1.98) |  | 1.542 (1.275-1.865) |  |
|  |  | Class3 | 2.745 (2.136-3.527) |  | 2.384 (1.929-2.947) |  | 2.372 (1.948-2.888) |  |
|  |  | Class4 | 4.585 (3.48-6.041) |  | 3.835 (2.991-4.917) |  | 3.728 (2.959-4.697) |  |
| Tea consumption |  |  |  | 0.351 |  | 0.532 |  | 0.159 |
| Never | 9361 | Class2 | 1.841 (1.521-2.227) |  | 1.764 (1.502-2.072) |  | 1.653 (1.393-1.962) |  |
|  |  | Class3 | 3.264 (2.639-4.037) |  | 2.627 (2.163-3.192) |  | 2.635 (2.201-3.155) |  |
|  |  | Class4 | 4.839 (3.76-6.228) |  | 3.731 (2.916-4.773) |  | 4.583 (3.671-5.721) |  |
| Occasionally or regularly | 9954 | Class2 | 1.717 (1.35-2.184) |  | 1.673 (1.361-2.056) |  | 1.611 (1.33-1.952) |  |
|  |  | Class3 | 2.751 (2.144-3.53) |  | 2.518 (2.022-3.136) |  | 2.401 (1.977-2.916) |  |
|  |  | Class4 | 4.306 (3.3-5.618) |  | 4.064 (3.196-5.167) |  | 3.735 (3.008-4.637) |  |
| High salt diet |  |  |  | 0.993 |  | 0.928 |  | 0.685 |
| No | 15166 | Class2 | 1.811 (1.538-2.133) |  | 1.712 (1.489-1.969) |  | 1.644 (1.425-1.897) |  |
|  |  | Class3 | 3.041 (2.545-3.633) |  | 2.518 (2.143-2.96) |  | 2.576 (2.22-2.988) |  |
|  |  | Class4 | 4.549 (3.718-5.565) |  | 3.846 (3.185-4.644) |  | 4.087 (3.434-4.865) |  |
| Yes | 4149 | Class2 | 1.814 (1.269-2.592) |  | 1.877 (1.398-2.522) |  | 1.694 (1.276-2.25) |  |
|  |  | Class3 | 3.082 (2.134-4.45) |  | 2.894 (2.112-3.967) |  | 2.448 (1.839-3.259) |  |
|  |  | Class4 | 4.897 (3.309-7.246) |  | 4.463 (3.134-6.357) |  | 4.114 (2.976-5.687) |  |
| High fat diet |  |  |  | 0.723 |  | 0.729 |  | 0.637 |
| No | 15750 | Class2 | 1.885 (1.604-2.215) |  | 1.735 (1.512-1.991) |  | 1.661 (1.443-1.912) |  |
|  |  | Class3 | 3.085 (2.587-3.677) |  | 2.51 (2.143-2.94) |  | 2.545 (2.199-2.945) |  |
|  |  | Class4 | 4.628 (3.796-5.642) |  | 3.932 (3.27-4.727) |  | 4.147 (3.495-4.92) |  |
| Yes | 3565 | Class2 | 1.514 (1.036-2.211) |  | 1.831 (1.329-2.522) |  | 1.674 (1.233-2.272) |  |
|  |  | Class3 | 2.875 (1.958-4.223) |  | 3.047 (2.161-4.295) |  | 2.58 (1.901-3.501) |  |
|  |  | Class4 | 4.652 (3.082-7.023) |  | 4.481 (3.05-6.584) |  | 4.09 (2.895-5.778) |  |
| High sugar diet |  |  |  | 0.123 |  | 0.016 |  | 0.205 |
| No | 15480 | Class2 | 1.772 (1.502-2.09) |  | 1.647 (1.428-1.899) |  | 1.611 (1.394-1.862) |  |
|  |  | Class3 | 2.86 (2.393-3.418) |  | 2.291 (1.948-2.694) |  | 2.377 (2.047-2.76) |  |
|  |  | Class4 | 4.218 (3.458-5.145) |  | 3.573 (2.968-4.301) |  | 3.89 (3.279-4.615) |  |
| Yes | 3835 | Class2 | 2.088 (1.487-2.933) |  | 2.18 (1.661-2.861) |  | 1.836 (1.4-2.407) |  |
|  |  | Class3 | 4.099 (2.862-5.869) |  | 4.113 (3.031-5.58) |  | 3.227 (2.444-4.26) |  |
|  |  | Class4 | 6.83 (4.577-10.195) |  | 5.797 (4.011-8.379) |  | 4.822 (3.421-6.795) |  |
| Diabetes or pre−diabetes |  |  |  | 0.008 |  | 0.089 |  | 0.001 |
| No | 16209 | Class2 | 1.951 (1.662-2.292) |  | 1.804 (1.575-2.066) |  | 1.706 (1.485-1.958) |  |
|  |  | Class3 | 3.479 (2.919-4.147) |  | 2.79 (2.383-3.267) |  | 2.715 (2.351-3.135) |  |
|  |  | Class4 | 5.499 (4.505-6.712) |  | 4.437 (3.679-5.352) |  | 4.631 (3.893-5.51) |  |
| Yes | 3106 | Class2 | 1.12 (0.755-1.663) |  | 1.376 (0.965-1.961) |  | 1.184 (0.842-1.665) |  |
|  |  | Class3 | 1.535 (1.03-2.287) |  | 1.802 (1.25-2.597) |  | 1.588 (1.135-2.221) |  |
|  |  | Class4 | 2.149 (1.414-3.265) |  | 2.546 (1.727-3.753) |  | 2.376 (1.673-3.372) |  |
| Hyperuricemia |  |  |  | 0.519 |  | 0.849 |  | 0.442 |
| No | 17451 | Class2 | 1.821 (1.566-2.118) |  | 1.74 (1.532-1.978) |  | 1.675 (1.47-1.908) |  |
|  |  | Class3 | 3.051 (2.589-3.595) |  | 2.607 (2.251-3.019) |  | 2.524 (2.204-2.89) |  |
|  |  | Class4 | 4.736 (3.931-5.707) |  | 3.935 (3.302-4.69) |  | 4.119 (3.503-4.844) |  |
| Yes | 1864 | Class2 | 1.277 (0.56-2.913) |  | 1.373 (0.577-3.269) |  | 1.147 (0.611-2.152) |  |
|  |  | Class3 | 2.244 (1.025-4.911) |  | 1.958 (0.846-4.531) |  | 2.104 (1.146-3.864) |  |
|  |  | Class4 | 3.222 (1.461-7.105) |  | 3.301 (1.418-7.685) |  | 3.234 (1.743-6.001) |  |
| Family history of CVD |  |  |  | 0.185 |  | 0.666 |  | 0.264 |
| No | 12130 | Class2 | 1.915 (1.572-2.335) |  | 1.82 (1.54-2.151) |  | 1.557 (1.325-1.829) |  |
|  |  | Class3 | 3.038 (2.462-3.75) |  | 2.62 (2.174-3.158) |  | 2.324 (1.968-2.743) |  |
|  |  | Class4 | 4.863 (3.865-6.12) |  | 4.052 (3.277-5.011) |  | 4.045 (3.337-4.902) |  |
| Yes | 7185 | Class2 | 1.717 (1.372-2.147) |  | 1.681 (1.386-2.039) |  | 1.872 (1.519-2.308) |  |
|  |  | Class3 | 3.139 (2.457-4.01) |  | 2.647 (2.115-3.314) |  | 3.003 (2.419-3.727) |  |
|  |  | Class4 | 4.225 (3.178-5.618) |  | 3.967 (3.037-5.183) |  | 4.179 (3.246-5.381) |  |

Table S5. Subgroup and interaction analyses among cumulative values of obesity- and lipid-related indices and CVD across various subgroups. Adjusted for gender, age, nationality, occupation, education, marital status, per capita monthly income, smoking status, alcohol consumption, physical exercise, tea consumption, high salt diet, high fat diet, high sugar diet, diabetes or pre-diabetes, hyperuricemia, family history of CVD, Scr, and AST/ALT., except in the corresponding group.

| Subgroup | N | Q | WHtR | | METS-VF | | CVAI | | BRI | | TyG | | TyG-WC | | TyG-BMI | |
| --- | --- | --- | --- | --- | --- | --- | --- | --- | --- | --- | --- | --- | --- | --- | --- | --- |
|  |  |  | OR (95% CI) | P for interaction | OR (95% CI) | P for interaction | OR (95% CI) | P for interaction | OR (95% CI) | P for interaction | OR (95% CI) | P for interaction | OR (95% CI) | P for interaction | OR (95% CI) | P for interaction |
| Sex |  |  |  | 0.826 |  | 0.476 |  | 0.215 |  | 0.869 |  | 0.275 |  | 0.909 |  | 0.825 |
| Male | 11292 | Q2 | 1.503 (1.188-1.902) |  | 1.694 (1.395-2.057) |  | 1.584 (1.174-2.138) |  | 1.479 (1.169-1.872) |  | 1.518 (1.257-1.832) |  | 1.821 (1.321-2.509) |  | 1.628 (1.32-2.009) |  |
|  |  | Q3 | 1.941 (1.551-2.428) |  | 2.212 (1.831-2.671) |  | 2.48 (1.866-3.296) |  | 1.952 (1.56-2.443) |  | 1.645 (1.37-1.975) |  | 2.363 (1.734-3.22) |  | 2.237 (1.83-2.735) |  |
|  |  | Q4 | 2.771 (2.221-3.456) |  | 3.278 (2.708-3.967) |  | 3.651 (2.751-4.847) |  | 2.743 (2.199-3.422) |  | 2.339 (1.954-2.8) |  | 3.815 (2.8-5.198) |  | 3.517 (2.878-4.297) |  |
| Female | 8023 | Q2 | 1.269 (1.079-1.493) |  | 1.495 (1.225-1.825) |  | 2.091 (1.764-2.479) |  | 1.256 (1.068-1.477) |  | 1.388 (1.165-1.655) |  | 1.672 (1.437-1.946) |  | 1.522 (1.271-1.822) |  |
|  |  | Q3 | 1.745 (1.458-2.09) |  | 1.647 (1.339-2.027) |  | 3.018 (2.45-3.718) |  | 1.742 (1.455-2.086) |  | 1.795 (1.497-2.151) |  | 2.277 (1.884-2.752) |  | 2.102 (1.742-2.537) |  |
|  |  | Q4 | 2.364 (1.926-2.902) |  | 2.781 (2.277-3.397) |  | 6.13 (4.688-8.015) |  | 2.364 (1.927-2.901) |  | 2.099 (1.708-2.579) |  | 3.309 (2.498-4.382) |  | 3.435 (2.805-4.205) |  |
| Age |  |  |  | 0.583 |  | 0.131 |  | 0.229 |  | 0.602 |  | 0.108 |  | <0.001 |  | <0.001 |
| >= 45 years | 6889 | Q2 | 1.352 (1.104-1.656) |  | 1.248 (0.958-1.626) |  | 1.468 (1.114-1.934) |  | 1.296 (1.059-1.585) |  | 1.381 (1.137-1.678) |  | 1.674 (1.366-2.052) |  | 1.308 (1.073-1.596) |  |
|  |  | Q3 | 1.642 (1.345-2.006) |  | 1.487 (1.16-1.907) |  | 2.197 (1.676-2.881) |  | 1.609 (1.32-1.963) |  | 1.64 (1.355-1.985) |  | 2.261 (1.829-2.796) |  | 1.761 (1.452-2.136) |  |
|  |  | Q4 | 2.321 (1.895-2.842) |  | 2.361 (1.859-2.997) |  | 3.212 (2.437-4.233) |  | 2.267 (1.854-2.773) |  | 1.969 (1.622-2.391) |  | 2.771 (2.204-3.485) |  | 2.379 (1.95-2.902) |  |
| < 45 years | 12426 | Q2 | 1.305 (1.098-1.551) |  | 1.661 (1.41-1.956) |  | 2.213 (1.859-2.635) |  | 1.318 (1.108-1.567) |  | 1.488 (1.255-1.765) |  | 1.736 (1.453-2.073) |  | 1.812 (1.503-2.185) |  |
|  |  | Q3 | 1.85 (1.547-2.211) |  | 2.022 (1.708-2.393) |  | 3.294 (2.682-4.046) |  | 1.888 (1.579-2.257) |  | 1.713 (1.441-2.037) |  | 2.206 (1.799-2.706) |  | 2.518 (2.085-3.041) |  |
|  |  | Q4 | 2.52 (2.101-3.023) |  | 3.057 (2.559-3.652) |  | 5.225 (4.186-6.521) |  | 2.524 (2.104-3.029) |  | 2.493 (2.087-2.98) |  | 4.359 (3.514-5.405) |  | 4.601 (3.806-5.563) |  |
| Nationality |  |  |  | 0.864 |  | 0.294 |  | 0.18 |  | 0.864 |  | 0.073 |  | 0.361 |  | 0.648 |
| Han nationality | 18716 | Q2 | 1.353 (1.186-1.544) |  | 1.593 (1.385-1.832) |  | 1.884 (1.629-2.178) |  | 1.337 (1.171-1.525) |  | 1.481 (1.302-1.685) |  | 1.747 (1.527-1.999) |  | 1.573 (1.372-1.805) |  |
|  |  | Q3 | 1.772 (1.55-2.024) |  | 1.916 (1.668-2.201) |  | 2.731 (2.343-3.182) |  | 1.78 (1.558-2.033) |  | 1.681 (1.478-1.913) |  | 2.248 (1.943-2.602) |  | 2.149 (1.876-2.461) |  |
|  |  | Q4 | 2.467 (2.154-2.825) |  | 2.949 (2.568-3.386) |  | 4.121 (3.505-4.844) |  | 2.449 (2.139-2.805) |  | 2.248 (1.971-2.564) |  | 3.484 (2.979-4.076) |  | 3.327 (2.898-3.82) |  |
| Others | 599 | Q2 | 1.051 (0.484-2.284) |  | 1.345 (0.572-3.162) |  | 1.297 (0.554-3.04) |  | 1.044 (0.481-2.27) |  | 0.569 (0.243-1.332) |  | 0.957 (0.423-2.167) |  | 1.57 (0.688-3.582) |  |
|  |  | Q3 | 1.797 (0.82-3.939) |  | 2.295 (0.987-5.333) |  | 1.882 (0.732-4.84) |  | 1.781 (0.812-3.905) |  | 1.671 (0.809-3.453) |  | 2.079 (0.865-4.998) |  | 2.151 (0.937-4.935) |  |
|  |  | Q4 | 2.945 (1.349-6.431) |  | 5.002 (2.151-11.632) |  | 6.421 (2.409-17.116) |  | 2.954 (1.351-6.459) |  | 1.602 (0.74-3.468) |  | 4.123 (1.606-10.586) |  | 5.008 (2.22-11.295) |  |
| Occupation |  |  |  | 0.441 |  | 1 |  | 0.685 |  | 0.348 |  | 0.888 |  | 0.51 |  | 0.118 |
| Woker | 15825 | Q2 | 1.4 (1.211-1.619) |  | 1.63 (1.4-1.897) |  | 1.983 (1.693-2.323) |  | 1.378 (1.192-1.594) |  | 1.502 (1.304-1.73) |  | 1.837 (1.584-2.131) |  | 1.716 (1.475-1.996) |  |
|  |  | Q3 | 1.923 (1.662-2.225) |  | 2.016 (1.734-2.343) |  | 2.877 (2.434-3.402) |  | 1.935 (1.673-2.239) |  | 1.754 (1.524-2.019) |  | 2.372 (2.021-2.784) |  | 2.403 (2.069-2.79) |  |
|  |  | Q4 | 2.622 (2.26-3.041) |  | 3.155 (2.715-3.668) |  | 4.395 (3.684-5.242) |  | 2.598 (2.241-3.014) |  | 2.309 (2-2.665) |  | 3.798 (3.199-4.509) |  | 3.746 (3.218-4.36) |  |
| Others | 3490 | Q2 | 1.136 (0.843-1.53) |  | 1.415 (1.012-1.979) |  | 1.357 (0.966-1.905) |  | 1.141 (0.847-1.537) |  | 1.221 (0.906-1.646) |  | 1.287 (0.953-1.737) |  | 1.103 (0.811-1.5) |  |
|  |  | Q3 | 1.199 (0.883-1.629) |  | 1.538 (1.104-2.141) |  | 1.96 (1.373-2.797) |  | 1.191 (0.877-1.619) |  | 1.401 (1.041-1.886) |  | 1.703 (1.218-2.382) |  | 1.29 (0.95-1.751) |  |
|  |  | Q4 | 1.9 (1.391-2.596) |  | 2.283 (1.643-3.172) |  | 2.97 (2.029-4.348) |  | 1.906 (1.396-2.602) |  | 1.888 (1.39-2.564) |  | 2.351 (1.637-3.378) |  | 2.089 (1.526-2.861) |  |
| Education |  |  |  | 0.003 |  | 0.617 |  | 0.009 |  | 0.002 |  | 0.011 |  | 0.001 |  | 0.005 |
| Middle school or below | 5667 | Q2 | 1.429 (1.144-1.784) |  | 1.484 (1.131-1.947) |  | 1.827 (1.4-2.383) |  | 1.383 (1.108-1.727) |  | 1.213 (0.984-1.495) |  | 1.999 (1.603-2.492) |  | 1.573 (1.257-1.967) |  |
|  |  | Q3 | 1.627 (1.301-2.034) |  | 1.713 (1.32-2.222) |  | 2.518 (1.921-3.299) |  | 1.62 (1.297-2.024) |  | 1.555 (1.268-1.908) |  | 2.297 (1.818-2.901) |  | 2.158 (1.734-2.686) |  |
|  |  | Q4 | 2.499 (1.997-3.127) |  | 2.75 (2.138-3.536) |  | 3.967 (2.998-5.25) |  | 2.468 (1.973-3.085) |  | 1.827 (1.48-2.255) |  | 3.393 (2.63-4.378) |  | 2.969 (2.37-3.719) |  |
| High school or equality | 5909 | Q2 | 1.106 (0.882-1.386) |  | 1.435 (1.123-1.834) |  | 1.47 (1.15-1.879) |  | 1.089 (0.869-1.365) |  | 1.671 (1.322-2.112) |  | 1.385 (1.098-1.748) |  | 1.318 (1.039-1.672) |  |
|  |  | Q3 | 1.556 (1.239-1.955) |  | 1.816 (1.427-2.311) |  | 2.091 (1.609-2.718) |  | 1.556 (1.24-1.953) |  | 1.67 (1.318-2.115) |  | 1.873 (1.449-2.421) |  | 1.905 (1.506-2.41) |  |
|  |  | Q4 | 2.042 (1.612-2.585) |  | 2.654 (2.084-3.379) |  | 3.362 (2.542-4.446) |  | 2.005 (1.584-2.538) |  | 2.358 (1.855-2.998) |  | 3.184 (2.42-4.19) |  | 3.061 (2.409-3.89) |  |
| College or above | 7739 | Q2 | 1.484 (1.176-1.874) |  | 1.685 (1.359-2.088) |  | 2.273 (1.783-2.896) |  | 1.504 (1.191-1.899) |  | 1.474 (1.179-1.844) |  | 1.738 (1.364-2.214) |  | 1.887 (1.475-2.414) |  |
|  |  | Q3 | 2.157 (1.703-2.731) |  | 2.055 (1.649-2.561) |  | 3.651 (2.781-4.793) |  | 2.186 (1.726-2.769) |  | 1.757 (1.402-2.201) |  | 2.611 (1.998-3.412) |  | 2.386 (1.862-3.058) |  |
|  |  | Q4 | 2.813 (2.211-3.578) |  | 3.184 (2.529-4.009) |  | 5.064 (3.786-6.774) |  | 2.834 (2.226-3.608) |  | 2.522 (2.005-3.171) |  | 3.888 (2.932-5.157) |  | 4.259 (3.318-5.468) |  |
| Marital status |  |  |  | 0.056 |  | 0.02 |  | 0.356 |  | 0.06 |  | 0.444 |  | 0.02 |  | 0.128 |
| Single | 2208 | Q2 | 1.216 (0.763-1.937) |  | 2.222 (1.504-3.284) |  | 2.029 (1.246-3.306) |  | 1.203 (0.755-1.917) |  | 1.571 (1.036-2.384) |  | 2.208 (1.35-3.612) |  | 1.373 (0.872-2.162) |  |
|  |  | Q3 | 1.906 (1.221-2.976) |  | 3.156 (2.09-4.764) |  | 2.918 (1.79-4.757) |  | 1.914 (1.225-2.99) |  | 2.146 (1.409-3.267) |  | 2.393 (1.397-4.099) |  | 2.052 (1.302-3.234) |  |
|  |  | Q4 | 3.347 (2.173-5.156) |  | 5.24 (3.238-8.479) |  | 4.393 (2.622-7.359) |  | 3.304 (2.144-5.091) |  | 2.503 (1.614-3.883) |  | 6.006 (3.503-10.298) |  | 4.544 (2.923-7.063) |  |
| Married | 17107 | Q2 | 1.339 (1.169-1.534) |  | 1.477 (1.274-1.712) |  | 1.795 (1.545-2.086) |  | 1.323 (1.155-1.515) |  | 1.423 (1.244-1.626) |  | 1.666 (1.451-1.912) |  | 1.569 (1.361-1.809) |  |
|  |  | Q3 | 1.734 (1.51-1.99) |  | 1.781 (1.54-2.06) |  | 2.628 (2.241-3.081) |  | 1.742 (1.518-1.999) |  | 1.633 (1.429-1.865) |  | 2.209 (1.901-2.566) |  | 2.129 (1.85-2.449) |  |
|  |  | Q4 | 2.379 (2.066-2.738) |  | 2.764 (2.394-3.192) |  | 4.031 (3.407-4.771) |  | 2.363 (2.053-2.72) |  | 2.183 (1.906-2.501) |  | 3.3 (2.806-3.88) |  | 3.229 (2.798-3.726) |  |
| Per capita monthly income |  |  |  | 0.301 |  | 0.499 |  | 0.919 |  | 0.359 |  | 0.896 |  | 0.743 |  | 0.045 |
| < 2000 yuan | 9946 | Q2 | 1.206 (1.004-1.449) |  | 1.47 (1.213-1.782) |  | 1.975 (1.616-2.414) |  | 1.204 (1.002-1.448) |  | 1.414 (1.184-1.69) |  | 1.668 (1.38-2.015) |  | 1.652 (1.364-2) |  |
|  |  | Q3 | 1.733 (1.444-2.079) |  | 1.795 (1.484-2.17) |  | 2.878 (2.328-3.557) |  | 1.762 (1.468-2.114) |  | 1.695 (1.423-2.02) |  | 2.341 (1.913-2.864) |  | 2.536 (2.104-3.057) |  |
|  |  | Q4 | 2.384 (1.981-2.868) |  | 2.888 (2.394-3.484) |  | 4.392 (3.516-5.487) |  | 2.393 (1.988-2.88) |  | 2.309 (1.93-2.764) |  | 3.574 (2.878-4.438) |  | 3.564 (2.945-4.314) |  |
| >= 2000yuan | 9369 | Q2 | 1.482 (1.232-1.783) |  | 1.704 (1.398-2.078) |  | 1.74 (1.417-2.136) |  | 1.448 (1.204-1.742) |  | 1.486 (1.238-1.783) |  | 1.753 (1.455-2.112) |  | 1.493 (1.233-1.808) |  |
|  |  | Q3 | 1.78 (1.472-2.152) |  | 2.057 (1.688-2.506) |  | 2.521 (2.03-3.131) |  | 1.765 (1.461-2.133) |  | 1.663 (1.384-1.999) |  | 2.112 (1.719-2.595) |  | 1.769 (1.459-2.145) |  |
|  |  | Q4 | 2.542 (2.095-3.084) |  | 3.053 (2.501-3.726) |  | 3.914 (3.109-4.927) |  | 2.496 (2.059-3.027) |  | 2.14 (1.773-2.581) |  | 3.362 (2.695-4.195) |  | 3.154 (2.596-3.831) |  |
| Smoking status |  |  |  | 0.756 |  | 0.535 |  | 0.328 |  | 0.797 |  | 0.34 |  | 0.105 |  | 0.775 |
| Not smoking or quitting smoking | 12068 | Q2 | 1.291 (1.113-1.496) |  | 1.59 (1.343-1.883) |  | 1.994 (1.705-2.332) |  | 1.28 (1.104-1.484) |  | 1.481 (1.277-1.718) |  | 1.692 (1.468-1.951) |  | 1.554 (1.326-1.821) |  |
|  |  | Q3 | 1.712 (1.468-1.996) |  | 1.823 (1.537-2.162) |  | 2.765 (2.321-3.295) |  | 1.721 (1.476-2.006) |  | 1.706 (1.467-1.985) |  | 2.304 (1.959-2.71) |  | 2.184 (1.862-2.561) |  |
|  |  | Q4 | 2.415 (2.055-2.838) |  | 2.962 (2.502-3.506) |  | 4.526 (3.722-5.503) |  | 2.414 (2.054-2.836) |  | 2.12 (1.805-2.491) |  | 3.143 (2.601-3.798) |  | 3.277 (2.775-3.871) |  |
| Yes | 7247 | Q2 | 1.549 (1.156-2.074) |  | 1.572 (1.235-2.001) |  | 1.472 (1.007-2.152) |  | 1.507 (1.126-2.017) |  | 1.394 (1.084-1.794) |  | 1.901 (1.253-2.885) |  | 1.605 (1.236-2.085) |  |
|  |  | Q3 | 2.006 (1.514-2.656) |  | 2.095 (1.66-2.645) |  | 2.462 (1.719-3.526) |  | 1.993 (1.508-2.635) |  | 1.654 (1.301-2.104) |  | 2.348 (1.567-3.517) |  | 2.101 (1.634-2.702) |  |
|  |  | Q4 | 2.776 (2.105-3.66) |  | 3.023 (2.389-3.823) |  | 3.553 (2.484-5.081) |  | 2.71 (2.058-3.567) |  | 2.352 (1.859-2.977) |  | 4.121 (2.755-6.165) |  | 3.513 (2.74-4.504) |  |
| Alcohol consumption |  |  |  | 0.532 |  | 0.197 |  | 0.444 |  | 0.523 |  | 0.499 |  | 0.63 |  | 0.846 |
| Not drinking or quitting drinking | 15436 | Q2 | 1.319 (1.147-1.517) |  | 1.613 (1.381-1.883) |  | 1.911 (1.642-2.224) |  | 1.304 (1.133-1.5) |  | 1.454 (1.266-1.67) |  | 1.72 (1.498-1.974) |  | 1.565 (1.351-1.812) |  |
|  |  | Q3 | 1.812 (1.572-2.089) |  | 1.966 (1.685-2.295) |  | 2.836 (2.409-3.339) |  | 1.817 (1.576-2.094) |  | 1.73 (1.506-1.988) |  | 2.27 (1.948-2.644) |  | 2.192 (1.895-2.535) |  |
|  |  | Q4 | 2.554 (2.206-2.957) |  | 3.184 (2.733-3.709) |  | 4.576 (3.836-5.459) |  | 2.546 (2.199-2.947) |  | 2.2 (1.904-2.542) |  | 3.413 (2.883-4.042) |  | 3.436 (2.958-3.992) |  |
| Yes | 3879 | Q2 | 1.392 (0.96-2.019) |  | 1.442 (1.061-1.96) |  | 1.45 (0.933-2.255) |  | 1.368 (0.942-1.985) |  | 1.435 (1.031-1.998) |  | 1.632 (0.959-2.776) |  | 1.585 (1.113-2.258) |  |
|  |  | Q3 | 1.562 (1.086-2.245) |  | 1.702 (1.264-2.293) |  | 1.954 (1.271-3.004) |  | 1.572 (1.094-2.259) |  | 1.505 (1.092-2.074) |  | 2.088 (1.239-3.518) |  | 1.933 (1.368-2.732) |  |
|  |  | Q4 | 2.149 (1.505-3.069) |  | 2.281 (1.679-3.098) |  | 2.712 (1.766-4.164) |  | 2.106 (1.474-3.009) |  | 2.241 (1.639-3.065) |  | 3.479 (2.065-5.864) |  | 3.041 (2.159-4.284) |  |
| Physical exercise |  |  |  | 0.314 |  | 0.245 |  | 0.195 |  | 0.368 |  | 0.465 |  | 0.202 |  | 0.806 |
| Never | 2896 | Q2 | 1.439 (0.988-2.098) |  | 1.474 (1.017-2.138) |  | 1.58 (1.053-2.371) |  | 1.394 (0.958-2.03) |  | 1.231 (0.857-1.767) |  | 1.963 (1.312-2.936) |  | 1.439 (0.995-2.081) |  |
|  |  | Q3 | 2.1 (1.444-3.055) |  | 2.281 (1.596-3.261) |  | 2.981 (1.982-4.483) |  | 2.044 (1.408-2.966) |  | 1.731 (1.217-2.461) |  | 3.211 (2.117-4.871) |  | 2.076 (1.449-2.974) |  |
|  |  | Q4 | 3.128 (2.152-4.548) |  | 3.302 (2.295-4.751) |  | 4.816 (3.142-7.382) |  | 3.049 (2.102-4.424) |  | 2.156 (1.508-3.082) |  | 5.143 (3.311-7.989) |  | 3.231 (2.258-4.623) |  |
| Occasionally | 8540 | Q2 | 1.409 (1.154-1.72) |  | 1.418 (1.159-1.736) |  | 2.03 (1.643-2.508) |  | 1.395 (1.143-1.704) |  | 1.436 (1.179-1.75) |  | 1.774 (1.447-2.174) |  | 1.736 (1.408-2.14) |  |
|  |  | Q3 | 1.87 (1.528-2.29) |  | 1.887 (1.546-2.303) |  | 2.937 (2.33-3.703) |  | 1.881 (1.537-2.302) |  | 1.76 (1.448-2.139) |  | 2.214 (1.773-2.766) |  | 2.334 (1.895-2.874) |  |
|  |  | Q4 | 2.375 (1.932-2.918) |  | 2.751 (2.25-3.364) |  | 4.189 (3.274-5.36) |  | 2.368 (1.927-2.91) |  | 2.15 (1.757-2.63) |  | 3.313 (2.608-4.208) |  | 3.506 (2.836-4.333) |  |
| Regularly | 7879 | Q2 | 1.263 (1.04-1.533) |  | 1.817 (1.454-2.271) |  | 1.786 (1.428-2.233) |  | 1.251 (1.031-1.519) |  | 1.541 (1.276-1.861) |  | 1.624 (1.336-1.974) |  | 1.477 (1.206-1.809) |  |
|  |  | Q3 | 1.598 (1.313-1.943) |  | 1.867 (1.494-2.334) |  | 2.438 (1.933-3.075) |  | 1.618 (1.33-1.968) |  | 1.612 (1.333-1.95) |  | 2.055 (1.66-2.544) |  | 2.017 (1.651-2.464) |  |
|  |  | Q4 | 2.382 (1.95-2.91) |  | 3.122 (2.507-3.888) |  | 3.91 (3.062-4.992) |  | 2.366 (1.937-2.89) |  | 2.323 (1.915-2.818) |  | 3.253 (2.584-4.094) |  | 3.248 (2.645-3.988) |  |
| Tea consumption |  |  |  | 0.979 |  | 0.347 |  | 0.144 |  | 0.962 |  | 0.031 |  | 0.82 |  | 0.308 |
| Never | 9361 | Q2 | 1.303 (1.1-1.544) |  | 1.631 (1.352-1.968) |  | 1.878 (1.559-2.263) |  | 1.301 (1.099-1.542) |  | 1.543 (1.292-1.843) |  | 1.713 (1.447-2.027) |  | 1.585 (1.322-1.901) |  |
|  |  | Q3 | 1.682 (1.41-2.005) |  | 1.759 (1.452-2.13) |  | 3 (2.451-3.673) |  | 1.677 (1.406-1.999) |  | 1.935 (1.62-2.312) |  | 2.202 (1.82-2.665) |  | 2.161 (1.8-2.594) |  |
|  |  | Q4 | 2.365 (1.968-2.843) |  | 2.892 (2.392-3.496) |  | 4.46 (3.565-5.58) |  | 2.369 (1.971-2.847) |  | 2.22 (1.837-2.682) |  | 3.414 (2.749-4.238) |  | 3.604 (2.983-4.354) |  |
| Occasionally or regularly | 9954 | Q2 | 1.395 (1.135-1.714) |  | 1.517 (1.236-1.861) |  | 1.767 (1.408-2.218) |  | 1.358 (1.105-1.669) |  | 1.343 (1.118-1.613) |  | 1.667 (1.34-2.073) |  | 1.522 (1.242-1.866) |  |
|  |  | Q3 | 1.857 (1.515-2.278) |  | 2.052 (1.683-2.503) |  | 2.345 (1.858-2.961) |  | 1.869 (1.525-2.291) |  | 1.453 (1.213-1.74) |  | 2.221 (1.77-2.787) |  | 2.071 (1.698-2.525) |  |
|  |  | Q4 | 2.62 (2.137-3.211) |  | 3.056 (2.506-3.727) |  | 3.754 (2.959-4.763) |  | 2.58 (2.105-3.161) |  | 2.156 (1.802-2.58) |  | 3.504 (2.771-4.431) |  | 3.147 (2.58-3.839) |  |
| High salt diet |  |  |  | 0.531 |  | 0.699 |  | 0.952 |  | 0.545 |  | 0.842 |  | 0.939 |  | 0.744 |
| No | 15166 | Q2 | 1.291 (1.117-1.493) |  | 1.584 (1.358-1.848) |  | 1.835 (1.567-2.149) |  | 1.276 (1.103-1.475) |  | 1.446 (1.252-1.669) |  | 1.686 (1.456-1.952) |  | 1.584 (1.361-1.843) |  |
|  |  | Q3 | 1.764 (1.523-2.043) |  | 1.856 (1.59-2.165) |  | 2.625 (2.218-3.107) |  | 1.77 (1.528-2.049) |  | 1.664 (1.441-1.921) |  | 2.195 (1.867-2.581) |  | 2.202 (1.893-2.56) |  |
|  |  | Q4 | 2.379 (2.046-2.767) |  | 2.941 (2.522-3.43) |  | 3.989 (3.333-4.774) |  | 2.36 (2.03-2.744) |  | 2.229 (1.924-2.582) |  | 3.374 (2.832-4.019) |  | 3.311 (2.837-3.863) |  |
| Yes | 4149 | Q2 | 1.525 (1.133-2.054) |  | 1.616 (1.182-2.211) |  | 1.961 (1.388-2.77) |  | 1.506 (1.118-2.028) |  | 1.443 (1.092-1.907) |  | 1.804 (1.321-2.463) |  | 1.539 (1.141-2.077) |  |
|  |  | Q3 | 1.78 (1.323-2.395) |  | 2.138 (1.583-2.887) |  | 3.05 (2.147-4.333) |  | 1.791 (1.331-2.41) |  | 1.738 (1.323-2.283) |  | 2.37 (1.719-3.267) |  | 1.974 (1.476-2.641) |  |
|  |  | Q4 | 2.806 (2.091-3.766) |  | 3.158 (2.338-4.266) |  | 4.771 (3.325-6.846) |  | 2.795 (2.082-3.752) |  | 2.213 (1.678-2.918) |  | 3.822 (2.728-5.354) |  | 3.534 (2.638-4.735) |  |
| High fat diet |  |  |  | 0.405 |  | 0.49 |  | 0.443 |  | 0.347 |  | 0.588 |  | 0.432 |  | 0.598 |
| No | 15750 | Q2 | 1.34 (1.161-1.546) |  | 1.595 (1.371-1.856) |  | 1.953 (1.671-2.283) |  | 1.322 (1.146-1.526) |  | 1.486 (1.289-1.712) |  | 1.738 (1.505-2.008) |  | 1.623 (1.399-1.883) |  |
|  |  | Q3 | 1.813 (1.568-2.095) |  | 1.981 (1.704-2.302) |  | 2.777 (2.349-3.282) |  | 1.824 (1.579-2.108) |  | 1.721 (1.494-1.984) |  | 2.143 (1.828-2.513) |  | 2.14 (1.845-2.483) |  |
|  |  | Q4 | 2.424 (2.089-2.812) |  | 2.951 (2.536-3.433) |  | 4.169 (3.49-4.98) |  | 2.407 (2.075-2.792) |  | 2.268 (1.96-2.623) |  | 3.408 (2.871-4.046) |  | 3.379 (2.903-3.932) |  |
| Yes | 3565 | Q2 | 1.337 (0.975-1.833) |  | 1.518 (1.083-2.127) |  | 1.449 (1.011-2.076) |  | 1.328 (0.969-1.82) |  | 1.312 (0.98-1.756) |  | 1.607 (1.146-2.253) |  | 1.388 (1-1.927) |  |
|  |  | Q3 | 1.582 (1.156-2.164) |  | 1.716 (1.232-2.39) |  | 2.374 (1.662-3.391) |  | 1.575 (1.151-2.155) |  | 1.556 (1.171-2.068) |  | 2.639 (1.865-3.734) |  | 2.208 (1.62-3.01) |  |
|  |  | Q4 | 2.643 (1.941-3.6) |  | 3.061 (2.22-4.219) |  | 4.022 (2.787-5.803) |  | 2.627 (1.928-3.579) |  | 2.108 (1.585-2.805) |  | 3.874 (2.689-5.58) |  | 3.39 (2.482-4.63) |  |
| High sugar diet |  |  |  | 0.023 |  | 0.13 |  | 0.027 |  | 0.031 |  | 0.386 |  | 0.032 |  | 0.28 |
| No | 15480 | Q2 | 1.234 (1.066-1.429) |  | 1.541 (1.322-1.796) |  | 1.883 (1.605-2.21) |  | 1.227 (1.06-1.42) |  | 1.446 (1.25-1.673) |  | 1.628 (1.402-1.891) |  | 1.531 (1.314-1.784) |  |
|  |  | Q3 | 1.651 (1.425-1.912) |  | 1.779 (1.528-2.072) |  | 2.604 (2.196-3.087) |  | 1.665 (1.438-1.928) |  | 1.601 (1.384-1.851) |  | 1.991 (1.691-2.344) |  | 2.019 (1.735-2.351) |  |
|  |  | Q4 | 2.224 (1.916-2.583) |  | 2.81 (2.414-3.272) |  | 3.823 (3.195-4.575) |  | 2.216 (1.909-2.574) |  | 2.139 (1.846-2.478) |  | 3.112 (2.614-3.706) |  | 3.165 (2.715-3.69) |  |
| Yes | 3835 | Q2 | 1.877 (1.407-2.503) |  | 1.836 (1.332-2.531) |  | 1.813 (1.317-2.496) |  | 1.805 (1.355-2.404) |  | 1.46 (1.124-1.898) |  | 2.082 (1.563-2.771) |  | 1.756 (1.315-2.344) |  |
|  |  | Q3 | 2.336 (1.736-3.142) |  | 2.731 (1.995-3.738) |  | 3.209 (2.31-4.459) |  | 2.303 (1.715-3.093) |  | 2.005 (1.549-2.597) |  | 3.409 (2.506-4.636) |  | 2.647 (1.997-3.509) |  |
|  |  | Q4 | 3.711 (2.749-5.011) |  | 3.87 (2.832-5.29) |  | 5.781 (4.073-8.207) |  | 3.626 (2.692-4.886) |  | 2.596 (1.974-3.415) |  | 5.168 (3.7-7.218) |  | 4.192 (3.12-5.632) |  |
| Diabetes or pre−diabetes |  |  |  | <0.001 |  | <0.001 |  | 0.013 |  | <0.001 |  | 0.187 |  | 0.076 |  | 0.004 |
| No | 16209 | Q2 | 1.38 (1.195-1.594) |  | 1.673 (1.438-1.947) |  | 1.995 (1.708-2.331) |  | 1.376 (1.191-1.589) |  | 1.387 (1.212-1.588) |  | 1.754 (1.521-2.023) |  | 1.617 (1.396-1.872) |  |
|  |  | Q3 | 1.932 (1.669-2.236) |  | 2.11 (1.813-2.455) |  | 3.089 (2.615-3.649) |  | 1.963 (1.696-2.272) |  | 1.669 (1.457-1.911) |  | 2.393 (2.043-2.803) |  | 2.259 (1.953-2.614) |  |
|  |  | Q4 | 2.788 (2.402-3.237) |  | 3.399 (2.921-3.954) |  | 4.791 (4.009-5.726) |  | 2.787 (2.4-3.236) |  | 2.209 (1.917-2.546) |  | 3.858 (3.251-4.577) |  | 3.675 (3.163-4.27) |  |
| Yes | 3106 | Q2 | 1.089 (0.795-1.49) |  | 1.068 (0.758-1.506) |  | 1.173 (0.804-1.711) |  | 1.036 (0.758-1.415) |  | 2.035 (1.346-3.078) |  | 1.411 (0.969-2.056) |  | 1.149 (0.801-1.65) |  |
|  |  | Q3 | 1.129 (0.831-1.532) |  | 1.083 (0.784-1.498) |  | 1.395 (0.96-2.028) |  | 1.085 (0.802-1.468) |  | 1.918 (1.292-2.847) |  | 1.536 (1.054-2.24) |  | 1.407 (0.996-1.988) |  |
|  |  | Q4 | 1.475 (1.088-2.001) |  | 1.541 (1.122-2.117) |  | 2.104 (1.442-3.071) |  | 1.421 (1.051-1.922) |  | 2.587 (1.767-3.789) |  | 2.198 (1.495-3.234) |  | 2.065 (1.475-2.89) |  |
| Hyperuricemia |  |  |  | 0.519 |  | 0.025 |  | 0.031 |  | 0.559 |  | 0.036 |  | 0.129 |  | 0.565 |
| No | 17451 | Q2 | 1.343 (1.175-1.534) |  | 1.542 (1.335-1.781) |  | 1.88 (1.625-2.175) |  | 1.326 (1.161-1.515) |  | 1.418 (1.244-1.617) |  | 1.679 (1.467-1.92) |  | 1.591 (1.386-1.827) |  |
|  |  | Q3 | 1.732 (1.512-1.984) |  | 1.867 (1.618-2.155) |  | 2.665 (2.279-3.115) |  | 1.743 (1.522-1.996) |  | 1.716 (1.507-1.955) |  | 2.268 (1.958-2.627) |  | 2.147 (1.871-2.464) |  |
|  |  | Q4 | 2.493 (2.17-2.865) |  | 3.021 (2.621-3.483) |  | 4.329 (3.668-5.11) |  | 2.476 (2.156-2.845) |  | 2.14 (1.869-2.451) |  | 3.417 (2.912-4.011) |  | 3.372 (2.928-3.883) |  |
| Yes | 1864 | Q2 | 1.209 (0.634-2.305) |  | 1.982 (1.193-3.291) |  | 1.093 (0.501-2.386) |  | 1.203 (0.631-2.295) |  | 1.922 (1.054-3.504) |  | 2.43 (0.893-6.617) |  | 1.09 (0.561-2.12) |  |
|  |  | Q3 | 1.862 (1.023-3.392) |  | 2.345 (1.429-3.846) |  | 2.024 (0.99-4.135) |  | 1.842 (1.012-3.355) |  | 1.523 (0.847-2.741) |  | 1.996 (0.753-5.292) |  | 1.675 (0.901-3.112) |  |
|  |  | Q4 | 2.265 (1.256-4.082) |  | 2.806 (1.707-4.615) |  | 2.383 (1.172-4.846) |  | 2.239 (1.242-4.037) |  | 2.893 (1.65-5.07) |  | 4.028 (1.536-10.561) |  | 2.669 (1.463-4.87) |  |
| Family history of CVD |  |  |  | 0.417 |  | 0.313 |  | 0.146 |  | 0.386 |  | 0.914 |  | 0.016 |  | 0.506 |
| No | 12130 | Q2 | 1.437 (1.209-1.708) |  | 1.611 (1.344-1.931) |  | 2.019 (1.67-2.442) |  | 1.422 (1.197-1.69) |  | 1.459 (1.24-1.716) |  | 1.918 (1.608-2.288) |  | 1.439 (1.213-1.709) |  |
|  |  | Q3 | 1.833 (1.543-2.177) |  | 2.068 (1.731-2.47) |  | 2.659 (2.179-3.245) |  | 1.832 (1.542-2.176) |  | 1.646 (1.4-1.935) |  | 2.222 (1.837-2.688) |  | 1.995 (1.685-2.362) |  |
|  |  | Q4 | 2.625 (2.206-3.124) |  | 3.09 (2.587-3.691) |  | 4.244 (3.451-5.221) |  | 2.609 (2.193-3.103) |  | 2.176 (1.845-2.567) |  | 3.795 (3.105-4.638) |  | 3.185 (2.684-3.779) |  |
| Yes | 7185 | Q2 | 1.243 (1.018-1.516) |  | 1.554 (1.255-1.925) |  | 1.691 (1.36-2.102) |  | 1.224 (1.003-1.494) |  | 1.434 (1.167-1.762) |  | 1.486 (1.213-1.819) |  | 1.855 (1.487-2.314) |  |
|  |  | Q3 | 1.72 (1.401-2.111) |  | 1.733 (1.398-2.149) |  | 2.839 (2.249-3.583) |  | 1.739 (1.417-2.134) |  | 1.752 (1.428-2.15) |  | 2.367 (1.897-2.955) |  | 2.473 (1.985-3.081) |  |
|  |  | Q4 | 2.269 (1.836-2.803) |  | 2.804 (2.263-3.475) |  | 4.012 (3.113-5.171) |  | 2.25 (1.821-2.781) |  | 2.325 (1.886-2.866) |  | 3.076 (2.404-3.935) |  | 3.728 (2.976-4.669) |  |

Table S6. Associations of changes in obesity- and lipid-related indices with CVD incidence.

|  | Model 1^a^ | | Model 2^b^ | | Model 3^c^ | |
| --- | --- | --- | --- | --- | --- | --- |
|  | OR (95% CI) | *p* | OR (95% CI) | *p* | OR (95% CI) | *p* |
| WHtR | | | | | | |
| Class 1 | Ref. |  | Ref. |  | Ref. |  |
| Class 2 | 1.127 (0.949 ~ 1.339) | 0.172 | 1.123 (0.945 ~ 1.335) | 0.189 | 1.067 (0.895 ~ 1.271) | 0.470 |
| Class 3 | 1.519 (1.252 ~ 1.842) | <.001 | 1.499 (1.234 ~ 1.821) | <.001 | 1.357 (1.110 ~ 1.659) | 0.003 |
| *P* for trend | <0.001 | | <0.001 | | 0.004 | |
| METS-VF |  | |  | |  | |
| Class 1 | Ref. |  | Ref. |  | Ref. |  |
| Class 2 | 1.443 (1.105 ~ 1.883) | 0.007 | 1.394 (1.067 ~ 1.821) | 0.015 | 1.325 (1.012 ~ 1.733) | 0.040 |
| Class 3 | 1.915 (1.466 ~ 2.501) | <.001 | 1.850 (1.415 ~ 2.419) | <.001 | 1.666 (1.267 ~ 2.189) | <.001 |
| *P* for trend | <0.001 | | <0.001 | | 0.003 | |
| CVAI |  | |  | |  | |
| Class 1 | Ref. |  | Ref. |  | Ref. |  |
| Class 2 | 1.128 (0.882 ~ 1.443) | 0.338 | 1.090 (0.851 ~ 1.397) | 0.496 | 1.048 (0.816 ~ 1.345) | 0.716 |
| Class 3 | 1.368 (1.057 ~ 1.769) | 0.017 | 1.329 (1.025 ~ 1.723) | 0.032 | 1.207 (0.925 ~ 1.575) | 0.166 |
| Class 4 | 1.846 (1.395 ~ 2.443) | <.001 | 1.792 (1.351 ~ 2.378) | <.001 | 1.553 (1.156 ~ 2.084) | 0.003 |
| *P* for trend | <0.001 | | <0.001 | | 0.003 | |
| BRI |  | |  | |  | |
| Class 1 | Ref. |  | Ref. |  | Ref. |  |
| Class 2 | 1.084 (0.919 ~ 1.280) | 0.338 | 1.080 (0.914 ~ 1.275) | 0.366 | 1.021 (0.863 ~ 1.209) | 0.807 |
| Class 3 | 1.462 (1.208 ~ 1.769) | <.001 | 1.446 (1.193 ~ 1.753) | <.001 | 1.301 (1.066 ~ 1.588) | 0.010 |
| *P* for trend | <0.001 | | <0.001 | | 0.003 | |
| TyG |  | |  | |  | |
| Class 1 | Ref. |  | Ref. |  | Ref. |  |
| Class 2 | 1.453 (1.241 ~ 1.700) | <.001 | 1.436 (1.226 ~ 1.682) | <.001 | 1.351 (1.149 ~ 1.587) | <.001 |
| Class 3 | 1.730 (1.433 ~ 2.090) | <.001 | 1.688 (1.395 ~ 2.043) | <.001 | 1.493 (1.216 ~ 1.834) | <.001 |
| *P* for trend | <0.001 | | <0.001 | | <0.001 | |
| TyG-WC |  | |  | |  | |
| Class 1 | Ref. |  | Ref. |  | Ref. |  |
| Class 2 | 1.294 (1.055 ~ 1.589) | 0.014 | 1.292 (1.051 ~ 1.587) | 0.015 | 1.246 (1.013 ~ 1.534) | 0.038 |
| Class 3 | 1.529 (1.222 ~ 1.914) | <.001 | 1.508 (1.203 ~ 1.891) | <.001 | 1.370 (1.084 ~ 1.732) | 0.008 |
| Class 4 | 2.277 (1.771 ~ 2.929) | <.001 | 2.237 (1.734 ~ 2.885) | <.001 | 1.948 (1.485 ~ 2.556) | <.001 |
| *P* for trend | <0.001 | | <0.001 | | <0.001 | |
| TyG-BMI |  | |  | |  | |
| Class 1 | Ref. |  | Ref. |  | Ref. |  |
| Class 2 | 1.354 (1.105 ~ 1.659) | 0.004 | 1.345 (1.097 ~ 1.649) | 0.004 | 1.296 (1.054 ~ 1.593) | 0.014 |
| Class 3 | 1.737 (1.417 ~ 2.128) | <.001 | 1.724 (1.405 ~ 2.115) | <.001 | 1.591 (1.286 ~ 1.968) | <.001 |
| Class 4 | 2.101 (1.659 ~ 2.661) | <.001 | 2.099 (1.654 ~ 2.664) | <.001 | 1.855 (1.438 ~ 2.394) | <.001 |
| *P* for trend | <0.001 | | <0.001 | | <0.001 | |

WHtR,waist-to-height ratio; METS-VF, metabolic score for visceral fat; CVAI, Chinese visceral adiposity index; BRI, body roundness index; TyG, triglyceride-glucose; TyG-WC, TyG-waist circumference; TyG-BMI, TyG-body mass index; OR, odds ratio; CI, confidence interval.

^a^ Adjusted for gender and age;

^b^Adjusted for gender, age, nationality, occupation, education, marital status, per capita monthly income, smoking status, alcohol consumption, physical exercise, tea consumption, high salt diet, high fat diet, and high sugar diet.

^c^Adjusted for variables in Model 2 and diabetes or pre-diabetes, hyperuricemia, family history of CVD, Scr, and AST/ALT.
